# Identification of network-based biomarkers of cardioembolic stroke using a systems biology approach with time series data

**DOI:** 10.1186/1752-0509-9-S6-S4

**Published:** 2015-12-09

**Authors:** Yung-Hao Wong, Chia-Chou Wu, Hsien-Yong Lai, Bo-Ren Jheng, Hsing-Yu Weng, Tzu-Hao Chang, Bor-Sen Chen

**Affiliations:** 1 Laboratory of Control and Systems Biology, Department of Electrical Engineering, National Tsing Hua University, Hsinchu 30013, Taiwan; 2 Institution Review Board, Christian Mennonite Hospital, Hualien 970, Taiwan; 3 Graduate Institute of Clinical Medicine, Taipei Medical University, Taipei 110, Taiwan; 4 Graduate Institute of Biomedical Informatics, Taipei Medical University, Taipei 110, Taiwan

**Keywords:** cardioembolic stroke, systems biology, network biomarker, protein-protein interaction

## Abstract

**Background:**

Molecular signaling of angiogenesis begins within hours after initiation of a stroke and the following regulation of endothelial integrity mediated by growth factor receptors and vascular growth factors. Recent studies further provided insights into the coordinated patterns of post-stroke gene expressions and the relationships between neurodegenerative diseases and neural function recovery processes after a stroke.

**Results:**

Differential protein-protein interaction networks (PPINs) were constructed at 3 post-stroke time points, and proteins with a significant stroke relevance value (SRV) were discovered. Genes, including *UBC, CUL3, APP, NEDD8, JUP*, and *SIRT7*, showed high associations with time after a stroke, and Ingenuity Pathway Analysis results showed that these post-stroke time series-associated genes were related to molecular and cellular functions of cell death, cell survival, the cell cycle, cellular development, cellular movement, and cell-to-cell signaling and interactions. These biomarkers may be helpful for the early detection, diagnosis, and prognosis of ischemic stroke.

**Conclusions:**

This is our first attempt to use our theory of a systems biology framework on strokes. We focused on 3 key post-stroke time points. We identified the network and corresponding network biomarkers for the 3 time points, further studies are needed to experimentally confirm the findings and compare them with the causes of ischemic stroke. Our findings showed that stroke-associated biomarker genes at different time points were significantly involved in cell cycle processing, including G_2_-M, G_1_-S and meiosis, which contributes to the current understanding of the etiology of stroke. We hope this work helps scientists reveal more hidden cellular mechanisms of stroke etiology and repair processes.

## Background

Stroke is the third leading cause of mortality and the primary cause of permanent disability worldwide; 87% of all strokes are ischemic [1]. Ischemic strokes are classified into cardioembolic, large-vessel, small-vessel lacunar, cryptogenic, and other causes based on stroke etiology. Cardiogenic embolisms account for ~20% of ischemic strokes each year [2]. Cardioembolic strokes are largely preventable through efforts at primary prevention for major-risk cardioembolic sources, e.g. high blood pressure, hyperlipidemia, etc. Once a cardioembolic stroke occurrs, the likelihood of recurrence is relatively high; therefore, the following prevention is also important. When known causes of strokes are identified, etiologic classification can guide treatments. Not knowing the etiology of a stroke restricts optimal therapy implementation and limits stroke research [3]. Several studies offered evidence of significant genetic implications in ischemic stroke [4]. We attempted to examine whether gene expression features in the blood can distinguish the causes of stroke, and determine whether these gene expression profiles can predict the stroke etiology and its outcomes.

Although no existing valid clinical criteria for diagnosing cardioembolic stroke, a diagnosis of cardioembolism can be based on the triad of (1) identification of a potential source of cardiogenic embolisms, (2) exclusion of other potential sources of cerebral ischemia, and (3) consideration of clinical neurologic features. Cardioembolism can be predicted on clinical grounds but is difficult to document [5]. Magnetic resonance imaging (MRI), echocardiography, Holter monitoring, transcranial Doppler, and electrophysiological studies increase the ability to identify the origin of cardioembolisms. In general, cardioembolic strokes have much worse prognosis and produce larger and more-disabling symptoms than other stroke subtypes. A recurrent embolism occurs in 30%~60% of patients with a history of a previous embolic event [6]. Cardioembolic strokes are a heterogeneous, complex disease resulting from interactions between genetic and environmental risk factors [7]. To understand contributions of various genetic risk factors to the etiology of stroke, the genetic risk factor must be analyzed and integrated in terms of biological functions and pathways [8]. With advances in affordable, high-throughput technologies, a systems biology study of diagnoses and treatments of cardioembolic strokes can shed light on applications of systems biology to the diagnosis, prognosis, and therapy of cardioembolic strokes.

In this study, we compared molecular interaction networks of 3 stages of cardioembolic strokes to reveal the underlying cellular mechanisms of cardioembolic strokes. As to different etiologies and heterogenic genomic alterations of cardioembolic stroke, the systems biology methodology integrated with Omics data is suitable to develop accurate diagnoses, novel therapeutic targets, and efficient targeted therapies. In this study, microarray data were applied to build the protein-protein interaction (PPI) networks (PPINs) of 3 stages of cardioembolic strokes. Network structures and protein association abilities in different stages of cardioembolic strokes were compared to obtain a set of significant proteins which can serve as important network biomarkers in the progressive process of cardioembolic strokes. In the future, significant proteins including *UBC, CUL3, APP, NEDD8, JUP, SIRT7*, etc., can be potent drug targets for first aid and emergency treatment within 24 h post-stroke. The complex behaviors of strokes differ from those of cancer and other complex diseases. We hope that this work can help scientists reveal more hidden cellular mechanisms of stroke etiology and repair processes.

## Materials and methods

### Overview of the construction process of stroke network marker

**Figure 1:**
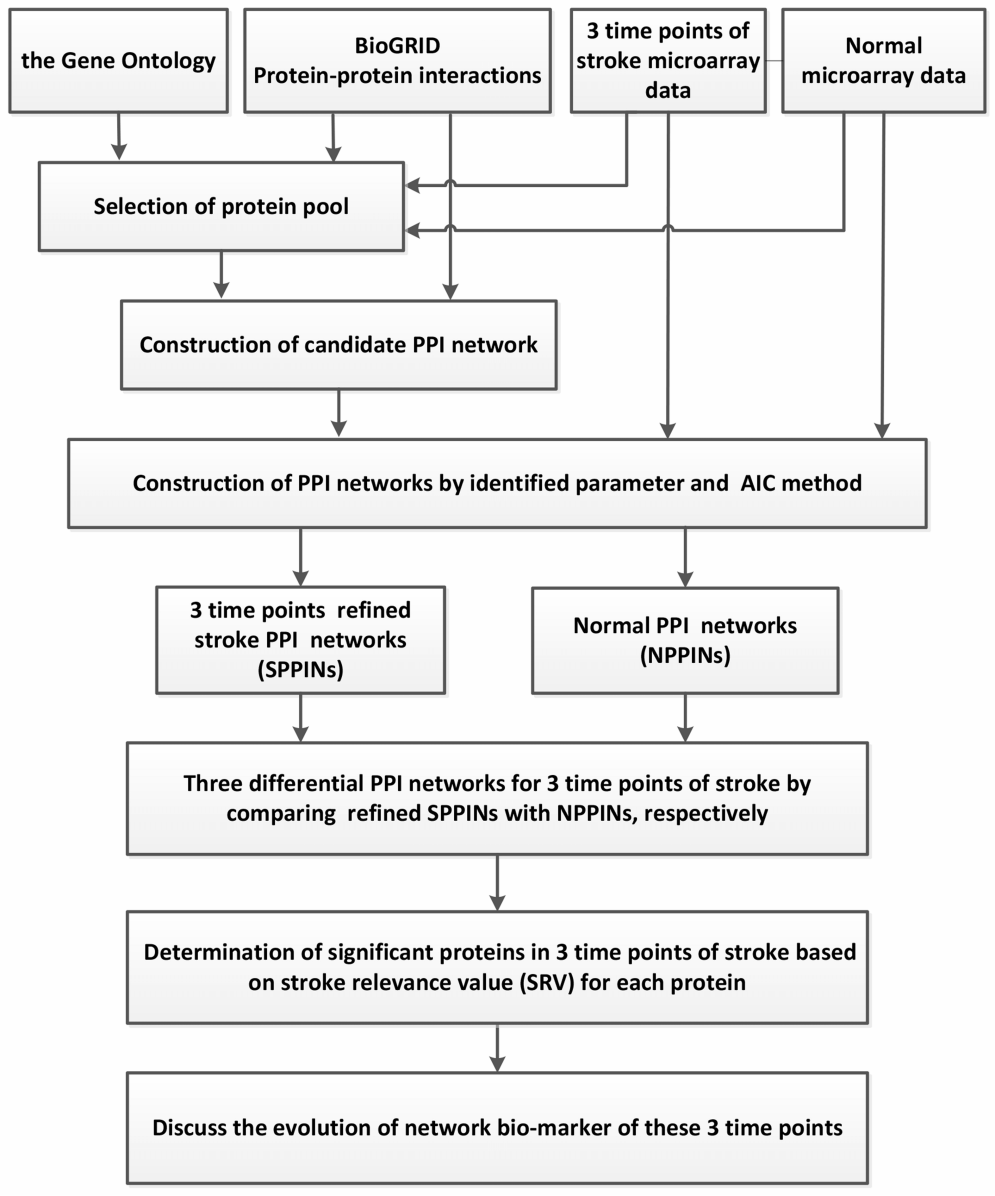
**Flowchart of constructing the network marker at 3 time points post-stroke**. We integrated microarray data, a gene ontology database, and protein-protein interaction (PPI) information to construct PPI networks (PPINs). These data were used for the differential protein pool selection, and then the selected proteins and their corresponding microarray data were used for the contribution of PPIN by a maximum-likelihood estimation and model order detection methods, resulting in a stroke PPIN (SPPIN) and a normal PPIN (NPPIN) in the 3 stages (3, 5, and 24 h post-stroke) of stroke. The 2 constructed PPINs were used to determine critical proteins of stroke by the difference of SPPIN and NPPIN matrices. By the help of the differential value of these two networks, the stroke relevance value (SRV) was computed for each protein, and significant proteins in the stroke recovery process were determined based on *p *values of the SRVs. These significant critical proteins with top SRVs were obtained as network markers for the 3 stages of stroke.

We successfully used our methods to find the core and specific network markers of 4 different cancers and the evolution of network markers from the early to late stages of bladder cancer [9,10]. A similar theoretical framework was employed in this study to find the evolution of network biomarkers of stroke at 3 time points which represent 3 important stages after a stroke has occurred. The theoretical systematic method in this paper was developed from a previous study. Figure 1 shows the flowchart to identify network biomarkers of stroke at 3 time points. Due to the theoretical framework have been successfully applied by us on various cancers and have been published on many journals, so we do not repeat it in detail in the main text. We only highlighted the significant key points of it and put the detailed description in the Additional file 1.

At first, two kinds of data sources were combined to build the network, they are microarray gene expression data and the protein-protein interaction data. We used them to construct the stroke PPINs (SPPINs, stroke protein-protein interaction networks) and normal PPIN (NPPINs). We calculated the stroke relevance value (SRV) for each protein in the network, and choose the proteins with top significant SRVs to be the network biomarkers. Detailed please refer to Additional file 1.

### Data sets selection and pre-processing

The stroke microarray dataset GSE58294 [11] and its corresponding platform, GPL570, were obtained from the NCBI GEO [12]. It contains gene expression data following a cardioembolic stroke. The dataset contained 3 time points of 23 stroke patients' samples and 23 control samples from non-disease subjects (totally 23*4 = 92 samples)(Table 1). We built 3 SPPINs for 3, 5, and 24 h post-stroke in this study and the NPPIN. We extract the PPI data for *Homo sapiens *from the online interaction repository with data compiled through comprehensive curation efforts, Biological General Repository for Interaction Database (BioGRID). It was used to delete false-positive PPIs for pruning PPINs. These PPINs of 3, 5, and 24 h post-stroke (3 SPPINs), and normal stage (NPPIN) were then compared mathematically to get SRVs and corresponding network markers (top SRVs). Detailed please refer to Additional file 1 [13-15].

**Table 1:** Descriptive information on datasets extracted from the GEO database used in this study.

Disease	GEO accession number	3 h Post-Stroke	5 h Post-Stroke	24 h Post-Stroke	Normal	platform
Stroke	GSE58294	23	23	23	23	GPL570

### Protein pool selection and the PPINs identification for stroke and normal samples

We collect a protein pool of those proteins with differential expressions to construct the corresponding SPPINs and NPPIN. A one-way analysis of variance (ANOVA) was used to screen out the differential proteins. We used the following protein association model to describe the PPI relationship:

(1) $$x_{i}\left(n\right)=\overset{M_{i}}{\underset{j=1}{\sum }}\alpha_{ij}x_{j}\left(n\right)+\omega_{i}\left(n\right)$$

where *x_i_*(*n*) is the target protein *i's *expression level for each sample *n *(stroke or normal); *x_j_*(*n*) is the *j*-th protein's expression level interacting with target protein *i *for each sample *n*; *α_ij _*means the ability of association interaction (combination strength) between the *i*-th target protein and its corresponding *j*-th interaction protein; *M_i _*is the number of proteins that interacting with their *i*-th target protein; and finally *ω_i_*(*n*) means stochastic noise caused by other factors in the biological systems or uncertainty of our model.

The second step is to use the maximum-likelihood (ML) estimation method [16] to determine associated parameters (combination strength) in (1) by the microarray expression data as follows (see Additional file 2):

(2) $$x_{i}\left(n\right)=\overset{M_{i}}{\underset{j=1}{\sum }}{\hat{\alpha }}_{ij}x_{j}\left(n\right)$$

where ${\hat{\alpha }}_{ij}$ was determined by using microarray expression data and the ML estimation method.

To do the model order selection and determine the significant protein interactions in${\hat{\alpha }}_{ij}$, finally we use the Akaike information criterion (AIC) [16] and a Student's *t*-test [17] method (see Additional file 3). Please refer to details in Additional file 1.

### Determination of the network structures and their corresponding significant proteins at 3, 5, and 24 h post-stroke and normal stage

After pruning away the spurious false-positive PPIs, only significant PPIs are remained:

(3) $$x_{i}\left(n\right)=\overset{M_{i}^{\prime }}{\underset{j=1}{\sum }}{\hat{\alpha }}_{ij}x_{j}\left(n\right),i=1,2.....M$$

where *M_i_*'≤*M_i _*is the number of significant PPIs in the total PPIN, with the *i*-th target protein. The refined PPIN is:

(4) X(n)=AX(n)+w(n)

where

$X\left(n\right)=\left[\begin{array}{c} x_{1}\left(n\right)\\ x_{2}\left(n\right)\\ \;\vdots \\ x_{M}\left(n\right) \end{array}\right]$, $A=\left[\begin{array}{ccc} {\hat{\alpha }}_{11} & \ldots & {\hat{\alpha }}_{1M}\\ \vdots & \ddots & \vdots \\ {\hat{\alpha }}_{M1} & \cdots \; & {\hat{\alpha }}_{MM} \end{array}\right]$, and $w\left(n\right)=\left[\begin{array}{c} {w_{1}}^{\text{'}}\left(n\right)\\ {w_{2}}^{\text{'}}\left(n\right)\\ \;\vdots \\ {w_{M}}^{\text{'}}\left(n\right) \end{array}\right]$

The interaction matrix *A *of refined PPINs in equation (4) for 3, 5, and 24 h post-stroke and normal cells was constructed, respectively, as follows:

(5) $$A_{S}^{k}=\left[\begin{array}{ccc} {\hat{\alpha }}_{11,S}^{k} & \ldots & {\hat{\alpha }}_{1M,S}^{k}\\ \vdots & \ddots & \vdots \\ {\hat{\alpha }}_{M1,S}^{k} & \cdots \; & {\hat{\alpha }}_{MM,S}^{k} \end{array}\right],\text{and}\;A_{N}^{}=\left[\begin{array}{ccc} {\hat{\alpha }}_{11,N}^{} & \ldots & {\hat{\alpha }}_{1M,N}^{}\\ \vdots & \ddots & \vdots \\ {\hat{\alpha }}_{M1,N}^{} & \cdots \; & {\hat{\alpha }}_{MM,N}^{} \end{array}\right]$$

where *k *= 3, 5, and 24 h post-stroke; $A_{S}^{k}$ and *A_N _*are the interaction matrices of the refined PPINs of 3, 5, and 24 h post-stroke, respectively; and *M *denotes the proteins number in the refined PPIN. The two protein association (combination strength) models for both SPPINs and the NPPIN for 3, 5, and 24 h post-stroke and normal stage are:

(6) $$\begin{array}{c} x_{S}^{k}\left(n\right)=A_{S}^{k}x_{S}\left(n\right)\\ x_{N}^{}\left(n\right)=A_{N}^{}x_{N}\left(n\right) \end{array}$$

where *k *= 3, 5, and 24 h post-stroke and $x_{S}^{k}\left(n\right)={\left[x_{1S}^{k}\;x_{2S}^{k}\;\cdots \;x_{MS}^{k}\right]}^{T}$ and *x_N_*(*n*)=[*x*_1*N *_*x*_2*N *_··· *x_MN_*]*^T ^*are vectors of proteins expression levels.

We defined the difference matrix $A_{S}^{k}-A_{N}^{}$ of the DPPIN between SPPINs and NPPIN as follows:

(7) $$D^{k}=\left[\begin{array}{ccc} d_{11}^{k} & \ldots & d_{1M}^{k}\\ \vdots & \ddots & \vdots \\ d_{M1}^{k} & \cdots \; & d_{MM}^{k} \end{array}\right]=\left[\begin{array}{ccc} {{\hat{\alpha }}^{k}}_{11,S}-{{\hat{\alpha }}^{}}_{11,N} & \ldots & {{\hat{\alpha }}^{k}}_{1M,S}-{{\hat{\alpha }}^{}}_{1M,N}\\ \vdots & \ddots & \vdots \\ {{\hat{\alpha }}^{k}}_{M1,S}-{{\hat{\alpha }}^{}}_{M1,N} & \cdots \; & {\hat{\alpha }}_{MM,S}^{k}-{\hat{\alpha }}_{MM,N}^{} \end{array}\right];$$

where *k *= 3, 5, and 24 h post-stroke; $d_{ij}^{k}$ is the protein association (combination strength) ability difference between SPPINs and NPPIN at *k *= 3, 5, and 24 h post-stroke and normal samples; and matrix *D^k ^*is the difference in network structures between SPPINs and the NPPIN for *k *= 3, 5, and 24 h post-stroke and normal samples.

Then we defined a stroke relevance value (SRV) to show the difference summation of SPPIN and NPPIN as follows [13]:

(8) $$SRV^{k}=\left[\begin{array}{c} SRV_{1}^{k}\\ \vdots \\ SRV_{i}^{k}\\ \vdots \\ SRV_{M}^{k} \end{array}\right]$$

where $SRV_{i}^{k}=\overset{M}{\underset{j=1}{\sum }}\left|d_{ij}^{k}\right|$, and *k *= 3, 5, and 24 h post-stroke. Detailed please refer to Additional file 1.

### Pathway analysis by many on-line freeware and powerful commercial software

We mapped the network biomarkers found to several on-line freeware of pathway analysis, such as KEEG (Kyoto Encyclopedia of Genes and Genomes) [18], NOA (network ontology analysis) [19,20] and the DAVID bioinformatics database [21,22]. They can help to investigate critical pathways related to these network markers and explore the relationships between these pathways and stroke. They also can illustrate the biological processes, cellular components and molecular functions. They also interpret the pathways involved in stoke etiology and repair processes. To complete our research results, we used the well-known commercial software, Ingenuity^® ^Pathway Analysis (IPA) and Metacore, to do multiple functional and pathway analyses. IPA^® ^is from QIAGEN (Redwood City, CA, http://www.qiagen.com/ingenuity). MetaCore™ is an integrated software suite from GeneGo for functional analysis of microarray, metabolic, SAGE, proteomics, siRNA, microRNA, and screening data. Please refer to details in Additional file 1.

## Results and discussion

### Evolution of network biomarkers at 3 post-stroke time points

**Figure 2:**
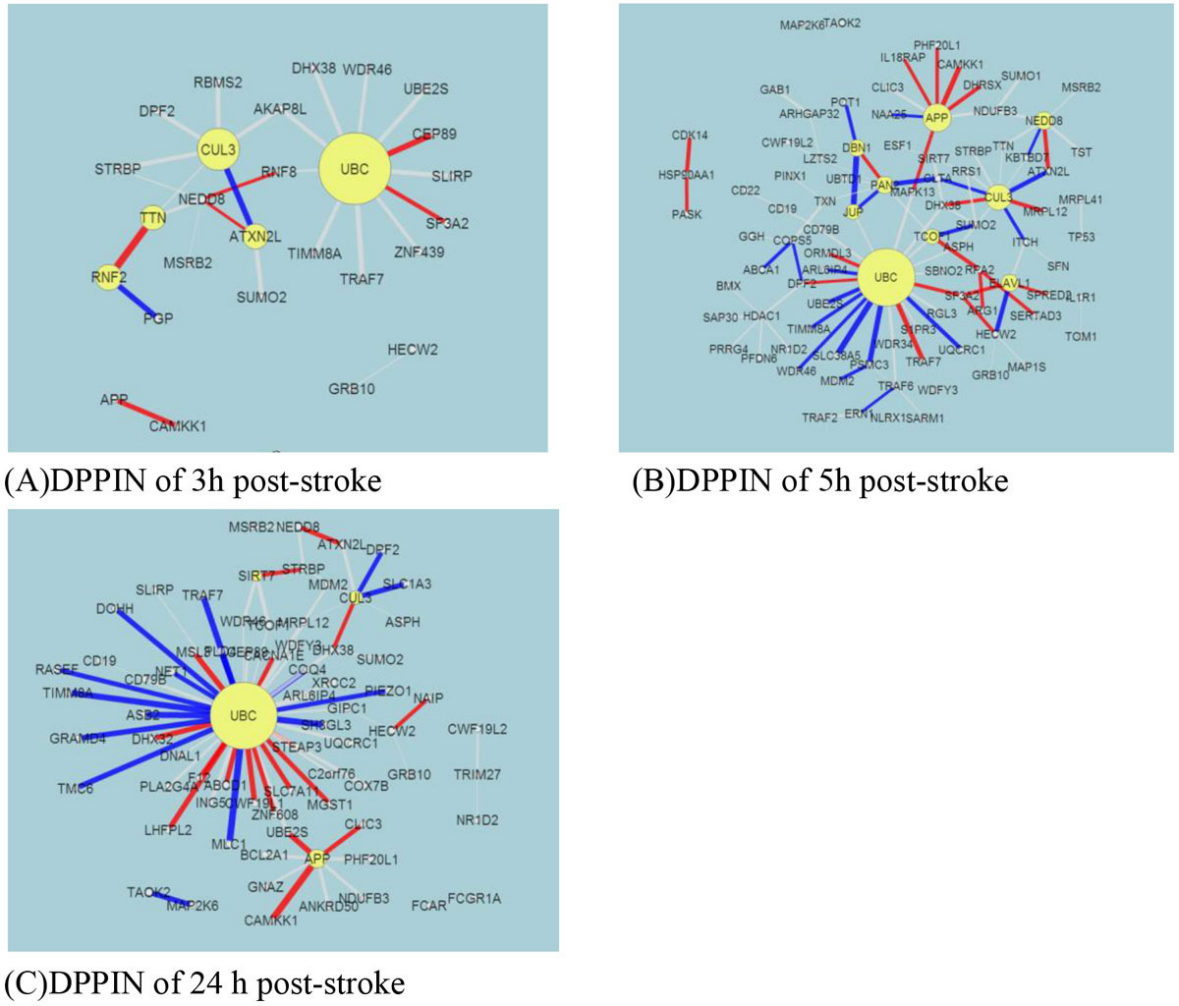
**The constructed differential protein-protein interaction (PPI) networks (PPINs; DPPINs) for 3 time points post-stroke**. This shows the DPPINs with edge and node information for 3 time points after a stroke occurred. It is the difference between the stroke PPIN (SPPIN) and normal PPIN (NPPIN). The node size means the stroke relevance value (SRV) of each protein, and the edge width is proportional to the link ability between the 2 proteins. Red and blue edges respectively indicate positive and negative values of *d_ij _*in (7). Besides *UBC*, we see at 3 h that *CUL3, ATXN2L, TTN*, and *NRF2 *dominate the network. At 5 h, *APP, CUL3, NEDD8, EVAL1, TCO, PAN*, and *JUP *dominate the network. At 24 h, *CLU3 *and *APP *dominate the network. We suggest that readers examine these figures together with Table 2. Information of the SRV and PPI are important for you to develop new therapeutic methods for stroke recovery. The figures were created using Cytoscape.

We built DPPINs for the 3 post-stroke time points (3, 5, and 24 h) (Figure 2). The SRVs of each protein in the 3 PPINs were calculated. One can find more information than SRVs in this figure, such as the edges and nodes of these PPINs. Screened by the *p *value of the SRV, we found significant proteins of network markers for these 3 stroke stages. Similar to our previous experience with bladder cancer [10], we wanted to reveal the repair mechanism of stroke at these 3 time points.

### Network markers at the 3 time points

After *p *value (≤0.01) screening, we found that there were 5, 9, and 4 significant proteins at 3, 5, and 24 h, respectively, post-stroke (Table 2). In addition, their corresponding SRVs respectively ranged 1.7~6.1, 2.1~11.7, and 1.7~26. These significant top SRV proteins and their corresponding PPIs were used to construct network markers at 3 post-stroke time points. We found that SRVs of stroke were much smaller than SRVs of our pervious cancer results [9,10], and also the cancer networks were much more complex than the stroke network. To compare the overall stroke process, we also combined samples at 3 time points into a total one (69 samples), and used it with normal data to build the DPPIN. This is not the main topic of this research, so we only put the total DPPIN in the results of Metacore. We do not discuss UBC in this paper, because it is another complex problem. It is a house keeping gene for many different kinds of diseases. We will extend our research on this target in the future.

**Table 2:** Top proteins at 3 time points post-stroke/

Protein	SRV	*p *value	Case_AvgExp	Control_AvgExp	Log2FC
**5 proteins at 3 h post-stroke **					

UBC [36]	6.07	<10^-9	18100	19375	-0.1
CUL3 [37]	3.26	0.001157	4178	3592	0.22
RNF2 [38]	1.77	0.031488	19	18	0.06
ATXN2L [39]	1.68	0.037603	70	164	-1.23
TTN [40]	1.65	0.041157	371	139	1.42

**9 proteins at 5 h post-stroke **					

UBC	11.69	<10^-9	18178	19375	-0.09
APP [41]	5	3.24E-05	1651	2018	-0.29
CUL3	4.44	0.000421	4486	3592	0.32
NEDD8 [42]	2.69	0.010615	3413	2952	0.21
PAN2 [43]	2.62	0.012395	293	491	-0.74
ELAVL1 [44]	2.53	0.014757	825	800	0.04
DBN1 [45]	2.37	0.021003	261	128	1.03
TCOF1 [46]	2.09	0.039515	109	229	-1.08
JUP [47]	2.06	0.042265	8	32	-2.04

**4 proteins at 24 h post-stroke **					

UBC	25.99	<10^-9	17540	19375	-0.14
APP	5.06	<10^-9	1653	2018	-0.29
CUL3	2.82	0.003012	4554	3592	0.34
SIRT7 [48]	1.66	0.042587	720	594	0.28

### Pathway analysis of network biomarkers at 3 h post-stroke

After SRV screening with our systems biology approach, the complete and complex functional and pathway analyses fundamentally revealed the evolutionary process of repair mechanisms of stroke. Because the number of significant proteins was very small compared to results for cancers, the KEGG results could not give us as much information as in cancer cases.

**Figure 3:**
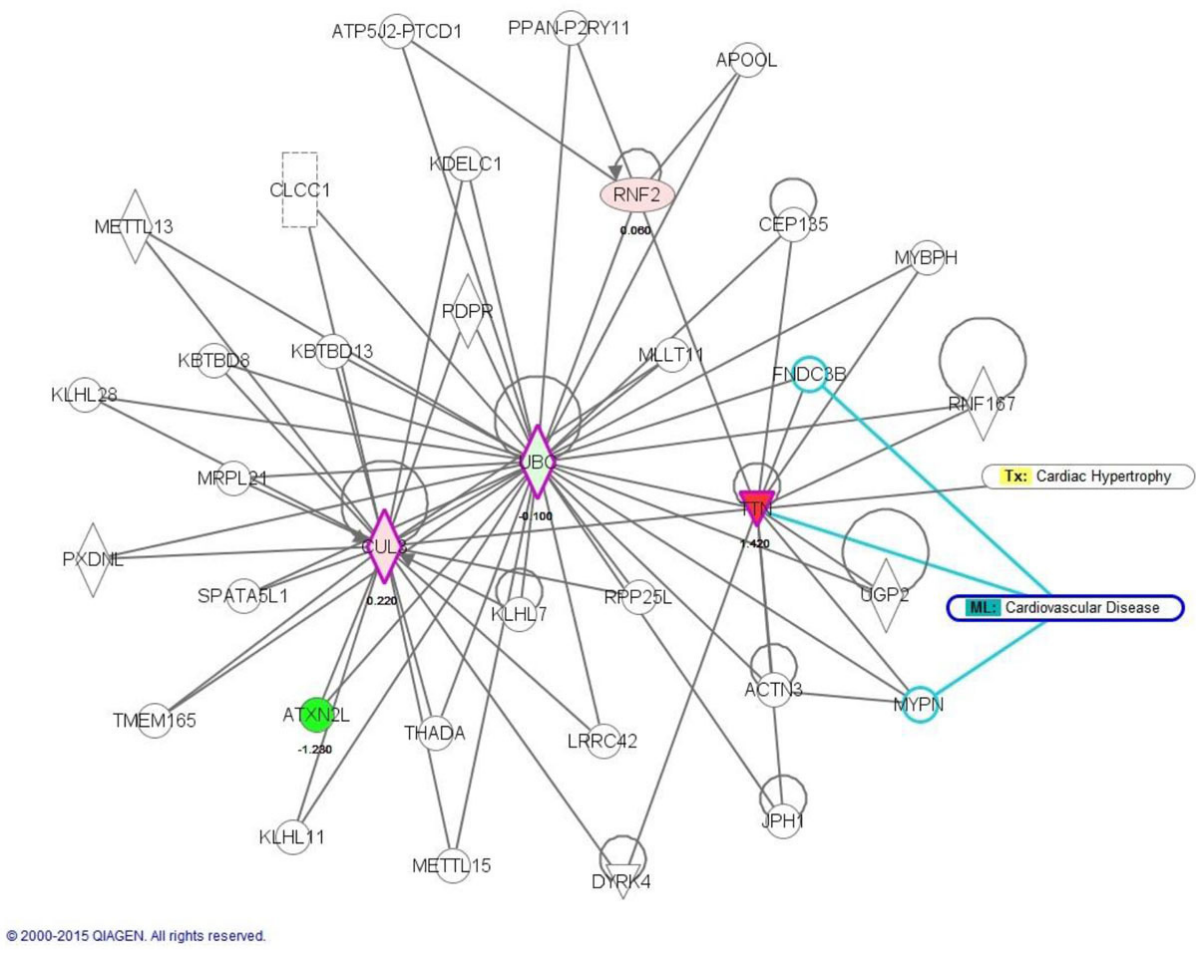
**IPA results at 3 h post-stroke**. Please refer to the legend of Figure 5 and 6.

The IPA gave us the clearest information on the disease, so we first show the IPA results (Table 3). We then show additional information given by NOA (Table 4). From Figure 3, one can see that the 2 key moduli of Tx_Cardiac-Hypertrophy and ML_Cardiovascular-Disease were related to our significant proteins (Figure 2(A)). We found that CUL3 appeared at all 3 stages, which implies that this time stationary network marker would be a significant target for therapy. It is easily seen that CUL3 is a key hub of the network. Functions and behaviors of CUL3 are very complex. Salinas et al. discussed how actinfilin acts as a CUL3 substrate adaptor, linking gluR6 kainate receptor subunits to the ubiquitin-proteasome pathway. They said that kainate receptors were implicated in excitotoxic neuronal death induced by stroke [23]. We list the disease functional analyses in a Additional file 3. The IPA results are shown in Table 3. NOA results are shown in Table 4. Results of Metacore are shown in Figure 7 to 14, for 3, 5, and 24 h, and the total (the sum of all samples).

**Table 3:** Functional analyses of the network biomarker at 3 h post- stroke.

Name	*p *value	Ratio
**Top canonical pathways**		

GABA receptor signaling	1.66E-02	1/67
Renal cell carcinoma signaling	1.75E-02	1/71
Toll-like receptor signaling	1.83E-02	1/74
Hereditary breast cancer signaling	2.83E-02	1/115
RhoA signaling	3.00E-02	1/122

**Top disease and biological functions**		

Cardiovascular disease	2.49E-04 ~ 3.94E-02	
Developmental disorder	2.49E-04 ~ 3.94E-02	
Hereditary disorder	2.49E-04 ~ 1.24E-03	
Organismal injury and abnormalities	2.49E-04 ~ 3.94E-02	
Skeletal and muscular disorders	2.49E-04 ~ 3.94E-02	

**Physiological system development and function**		

Embryonic development	6.94E-05 ~ 4.35E-02	
Organismal development	9.92E-05 ~ 4.35E-02	
Cardiovascular system development and function	4.98E-04 ~ 3.85E-02	
Hematological system development and function	4.98E-04 ~ 1.48E-02	
Hepatic system development and function	4.98E-04 ~ 3.00E-02	

**Table 4:** Pathway analysis and gene set enrichment analysis of 5 proteins at 3 h post-stroke on (1) biological processes, (2) cellular components and (3) molecular functions by NOA.

GO: term	*p *value	Corrected *p *value	R	T	G	O	Term name
**(1) Biological Processes**							

GO:0042787	0.0033	0.0825	6357	1	21	1	protein ubiquitination involved in ubiquitin-dependent protein catabolic process
GO:0016567	0.0135	0.3382	6357	1	86	1	protein ubiquitination
GO:0032446	0.0171	0.4286	6357	1	109	1	protein modification by small protein conjugation
GO:0070647	0.0215	0.5387	6357	1	137	1	protein modification by small protein conjugation or removal
GO:0019941	0.0272	0.6803	6357	1	173	1	modification-dependent protein catabolic process
GO:0006511	0.0272	0.6803	6357	1	173	1	ubiquitin-dependent protein catabolic process
GO:0051603	0.0289	0.7236	6357	1	184	1	proteolysis involved in cellular protein catabolic process
GO:0043632	0.03	0.7511	6357	1	191	1	modification-dependent macromolecule catabolic process
GO:0006508	0.047	1	6357	1	299	1	proteolysis

**(2) Cellular Components**							

GO:0031463	7.8E-4	0.0110	6357	1	5	1	Cul3-RING ubiquitin ligase complex
GO:0031461	0.0042	0.0594	6357	1	27	1	cullin-RING ubiquitin ligase complex
GO:0000151	0.0099	0.1387	6357	1	63	1	ubiquitin ligase complex

**(3) Molecular Functions**							

GO:0031625	0.0020	0.0224	6357	1	13	1	ubiquitin protein ligase binding
GO:0019899	0.0056	0.0622	6357	1	36	1	enzyme binding
GO:0004842	0.0133	0.1470	6357	1	85	1	ubiquitin-protein ligase activity
GO:0019787	0.0143	0.1574	6357	1	91	1	small conjugating protein ligase activity
GO:0016881	0.0157	0.1730	6357	1	100	1	acid-amino acid ligase activity
GO:0016879	0.0212	0.2336	6357	1	135	1	ligase activity, forming carbon-nitrogen bonds
GO:0016874	0.0309	0.3408	6357	1	197	1	ligase activity

R: number of genes in the reference set. T: number of genes in the test set. G: number of genes annotated by a given term in the reference set. O: number of genes annotated by a given term in the test set.

### Pathway analysis of network biomarkers at 5 h post-stroke

**Figure 4:**
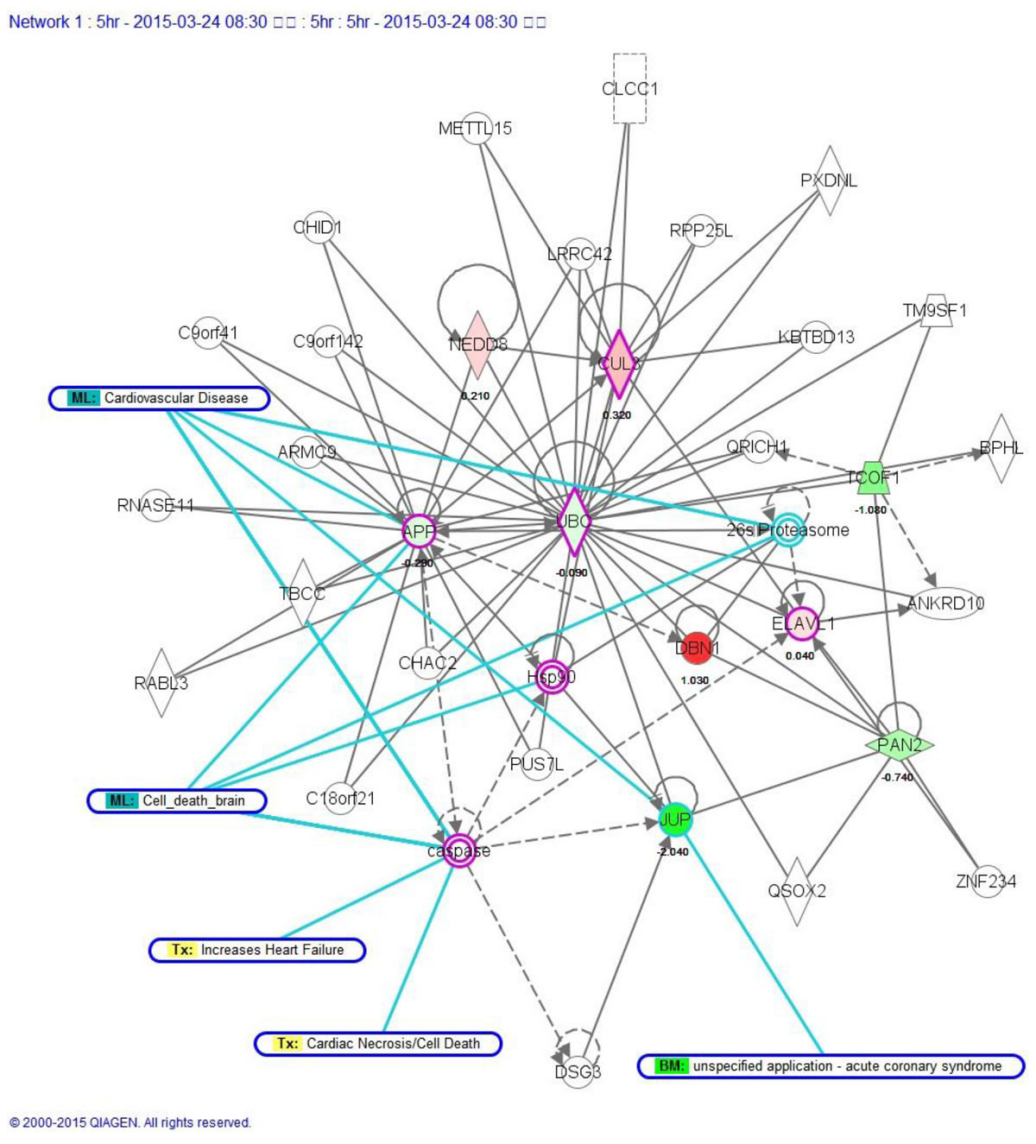
**IPA results at 5 h post-stroke**. Please refer to the legend of Figure 5 and 6.

IPA results (Figure 4) show that there were 5 modules of ML_Cardiovascular-Disease, ML_Cell-Death-Brain, Tx_Increases-Heart-Failure, Tx_Cardiac Necrosis/Cell Death, and BM_Unspecified-Application/Actute-Coronary Syndrome related to our significant proteins (Figure 2(B)). We found that caspase was related to 4 modules. Aries et el. discussed caspase-1 cleavage of transcription factor GATA4 and regulation of cardiac cell fate. They showed that GATA4 is cleaved by caspase-1 in cardiomyocytes, and their data identified a target for caspase-1 in nuclei and a pathway to explain its related cardiac actions [24]. The amyloid precursor protein (APP) is part of a binding-protein-dependent transport system. It is probably responsible for translocation of substrate across membranes, and it belongs to the permease family of the binding-protein-dependent transport system. It is also known as the β-amyloid (Aβ) precursor protein. From [25], we know that APP is a key gene related to Alzheimer disease (AD), and it implicates the relationship between neurodegenerative diseases and stroke. A lot of research has discussed this gene [26-29]. It could possibly be an efficient therapy target at this time point. We list the disease functional analyses in a Additional file 3. IPA results are shown in Table 5. NOA results are shown in Table 6.

**Table 5:** Functional analyses of the network biomarker at 5 h post-stroke.

Name	*p *value	Ratio
**Top canonical pathways**		

Protein ubiquitination pathway	5.44E-03	2/255
Docosahexaenoic acid (DHA) signaling	1.73E-02	1/39
Neuroprotective role of THOP1 in Alzheimer's disease	1.78E-02	1/40
Amyloid processing	2.26E-02	1/51
GABA receptor signaling	2.96E-02	1/67

**Top disease and biological functions**		

Cancer	4.48E-04 ~ 7.14E-03	
Cardiovascular disease	4.48E-04 ~ 5.36E-03	
Connective tissue disorders	4.48E-04 ~ 2.68E-03	
Developmental disorder	4.48E-04 ~ 7.14E-03	
Hematological disease	4.48E-04 ~ 4.47E-03	

**Physiological system development and function**		

Embryonic development	3.80E-06 ~ 8.48E-03	
Organismal development	3.80E-06 ~ 8.48E-03	
Tissue morphology	3.80E-06 ~ 8.03E-03	
Organ morphology	6.22E-05 ~ 8.48E-03	
Reproductive system development and function	6.22E-05 ~ 6.70E-03	

**Table 6:** Pathway analysis and gene set enrichment analysis of 9 proteins at 5 h post-stroke on (1) biological processes, (2) cellular components and (3) molecular functions by NOA.

GO: term	*p *value	Corrected *p *value	R	T	G	O	Term name
**(1) Biological Processes**							

GO:0019941	7.3E-4	0.0316	6357	2	173	2	modification-dependent protein catabolic process
GO:0006511	7.3E-4	0.0316	6357	2	173	2	ubiquitin-dependent protein catabolic process
GO:0051603	8.3E-4	0.0358	6357	2	184	2	proteolysis involved in cellular protein catabolic process
GO:0043632	8.9E-4	0.0386	6357	2	191	2	modification-dependent macromolecule catabolic process
GO:0006508	0.0022	0.0948	6357	2	299	2	proteolysis
GO:0044265	0.0039	0.1698	6357	2	400	2	cellular macromolecule catabolic process
GO:0009057	0.0044	0.1899	6357	2	423	2	macromolecule catabolic process
GO:0006301	0.0059	0.2566	6357	2	19	1	postreplication repair
GO:0042787	0.0065	0.2836	6357	2	21	1	protein ubiquitination involved in ubiquitin-dependent protein catabolic process
GO:0044248	0.0106	0.4600	6357	2	658	2	cellular catabolic process

**(2) Cellular Components**							

GO:0031251	6.2E-4	0.0106	6357	2	2	1	PAN complex
GO:0031463	0.0015	0.0267	6357	2	5	1	Cul3-RING ubiquitin ligase complex
GO:0031461	0.0084	0.1441	6357	2	27	1	cullin-RING ubiquitin ligase complex
GO:0000151	0.0197	0.3353	6357	2	63	1	ubiquitin ligase complex
GO:0043234	0.0463	0.7879	6357	2	1369	2	protein complex

**(3) Molecular Functions**							

GO:0004535	0.0012	0.0314	6357	2	4	1	poly(A)-specific ribonuclease activity
GO:0031625	0.0040	0.1021	6357	2	13	1	ubiquitin protein ligase binding
GO:0000175	0.0053	0.1335	6357	2	17	1	3'-5'-exoribonuclease activity
GO:0004221	0.0059	0.1492	6357	2	19	1	ubiquitin thiolesterase activity
GO:0016896	0.0065	0.1649	6357	2	21	1	exoribonuclease activity, producing 5'-phosphomonoesters
GO:0004532	0.0065	0.1649	6357	2	21	1	exoribonuclease activity
GO:0016790	0.0084	0.2119	6357	2	27	1	thiolester hydrolase activity
GO:0008408	0.0084	0.2119	6357	2	27	1	3'-5' exonuclease activity
GO:0016796	0.0097	0.2432	6357	2	31	1	exonuclease activity, active with either ribo- or deoxyribonucleic acids and producing 5'-phosphomonoesters
GO:0019899	0.0112	0.2823	6357	2	36	1	enzyme binding

R: number of genes in the reference set. T: number of genes in the test set. G: number of genes annotated by a given term in the reference set. O: number of genes annotated by a given term in the test set.

### Pathway analysis of network biomarkers at 24 h post-stroke

**Figure 5:**
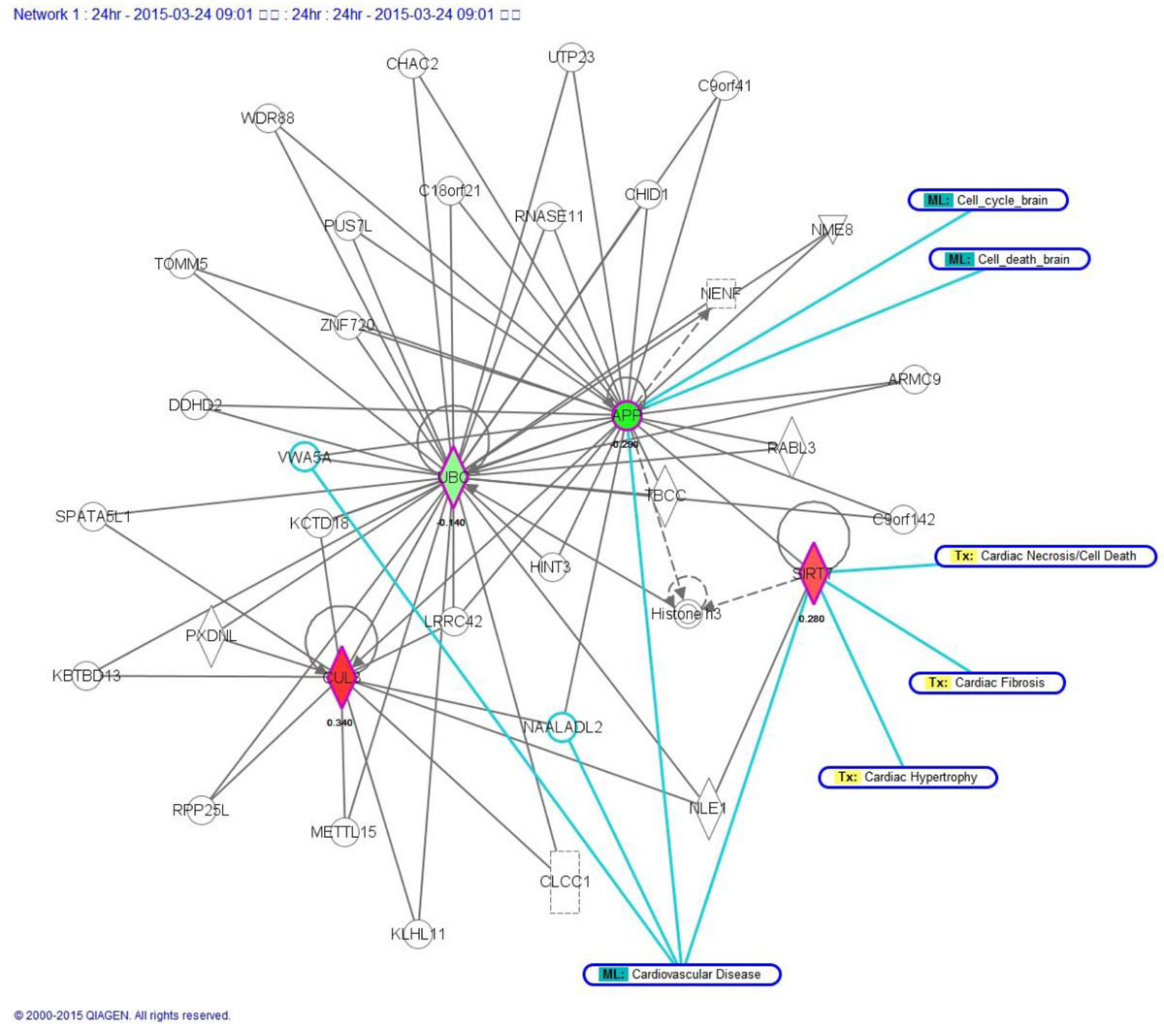
**IPA results at 24 h post-stroke**. By the IPA analysis, one can see that the 3 network markers are related to different modules at 3 different time points (3, 5, and 24 h) post-stroke. It is easy to see the evolutionary process of network biomarkers. From the detailed legend in Figure 6, one can see different regulatory mechanisms at these 3 time points of stroke. This abundant information can offer experts various novel strategies to develop stroke therapies or recovery methods. The experts can decide to inhibit or activate key proteins in these networks. And experts can refer to a patient's medical history to decide the therapeutic strategy. We analyzed the stroke relevance value (SRV) results by IPA software, and it gave us more clues to uncover hidden mechanisms of stroke. We consider this inspired pioneering work, and in the future, experts need to design new therapies or recovery strategies for validation.

**Figure 6:**
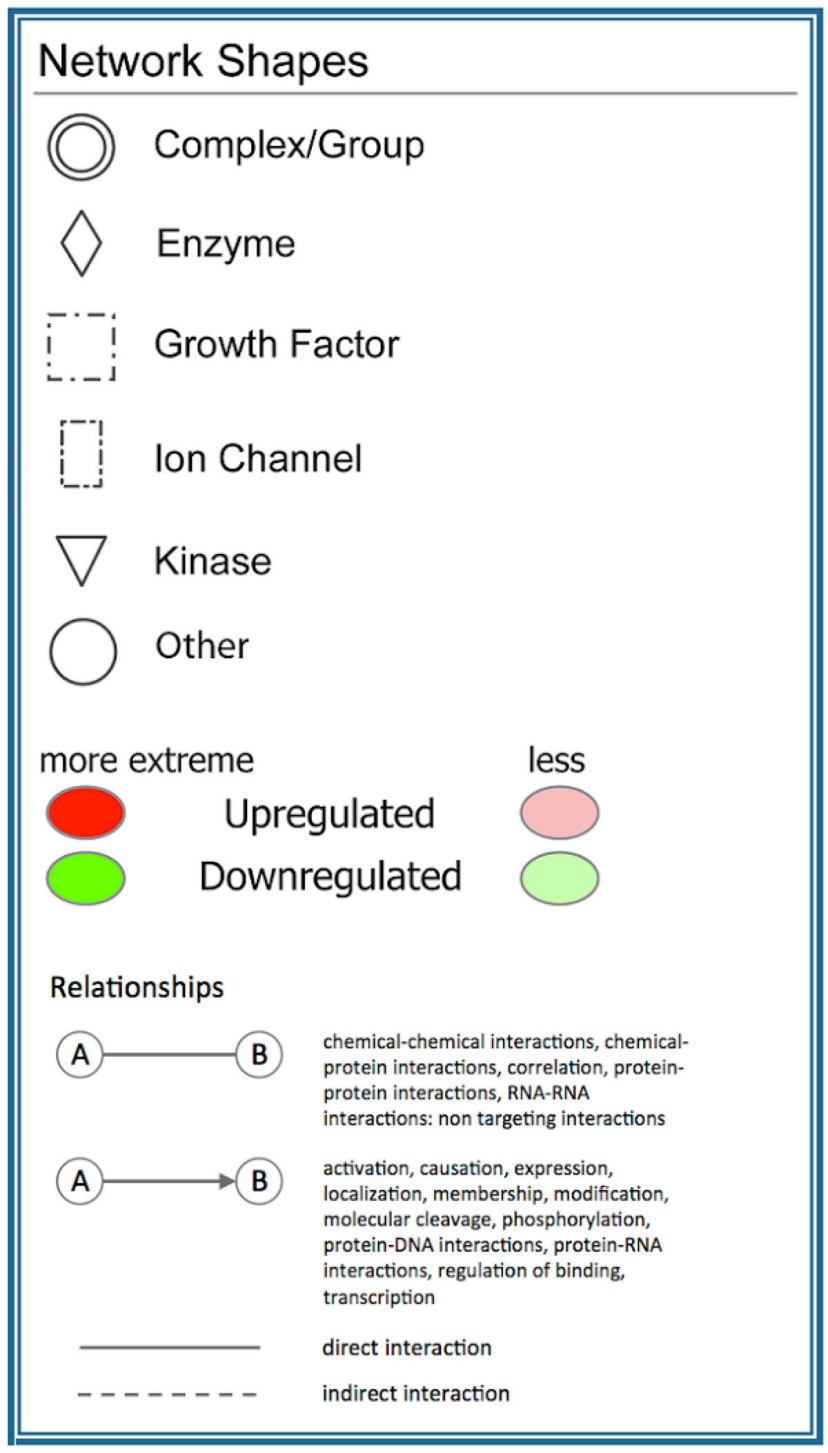
**The detailed legend of IPA in Figures 3 to 5**.

**Figure 7:**
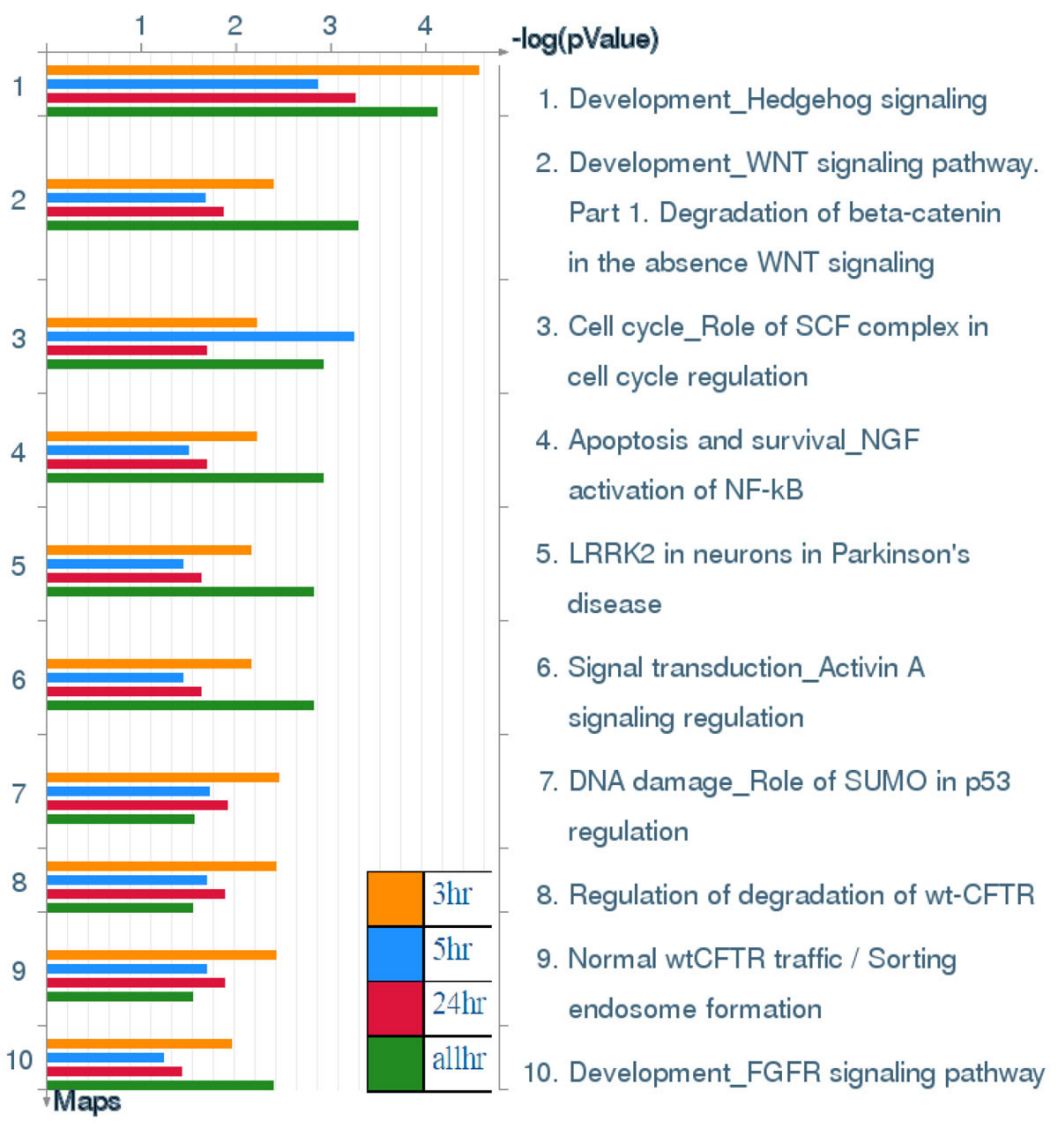
**Pathway maps of Metacore**. Sorting is done for the 'Statistically significant Maps'. Canonical pathway maps represent a set of signaling and metabolic maps covering human in a comprehensive way. All maps are created by Thomson Reuters scientists by a high-quality manual curation process based on published peer-reviewed literature. [The above paragraph is directly cited from the Metacore results.]. Figure 7-14 are serial maps generated by Metacore should give experts more choices and strategies to attack the core network post-stroke. Figure 7-12 show pathway maps for the 3 time points of stroke. Figure 13 shows the process networks. We can see cell cycle G2-M, G1-S, and meiosis are the top 3 process networks. They give experts actual targets to develop novel strategies. Figure 14 shows our network markers related to statistically significant diseases.

**Figure 8:**
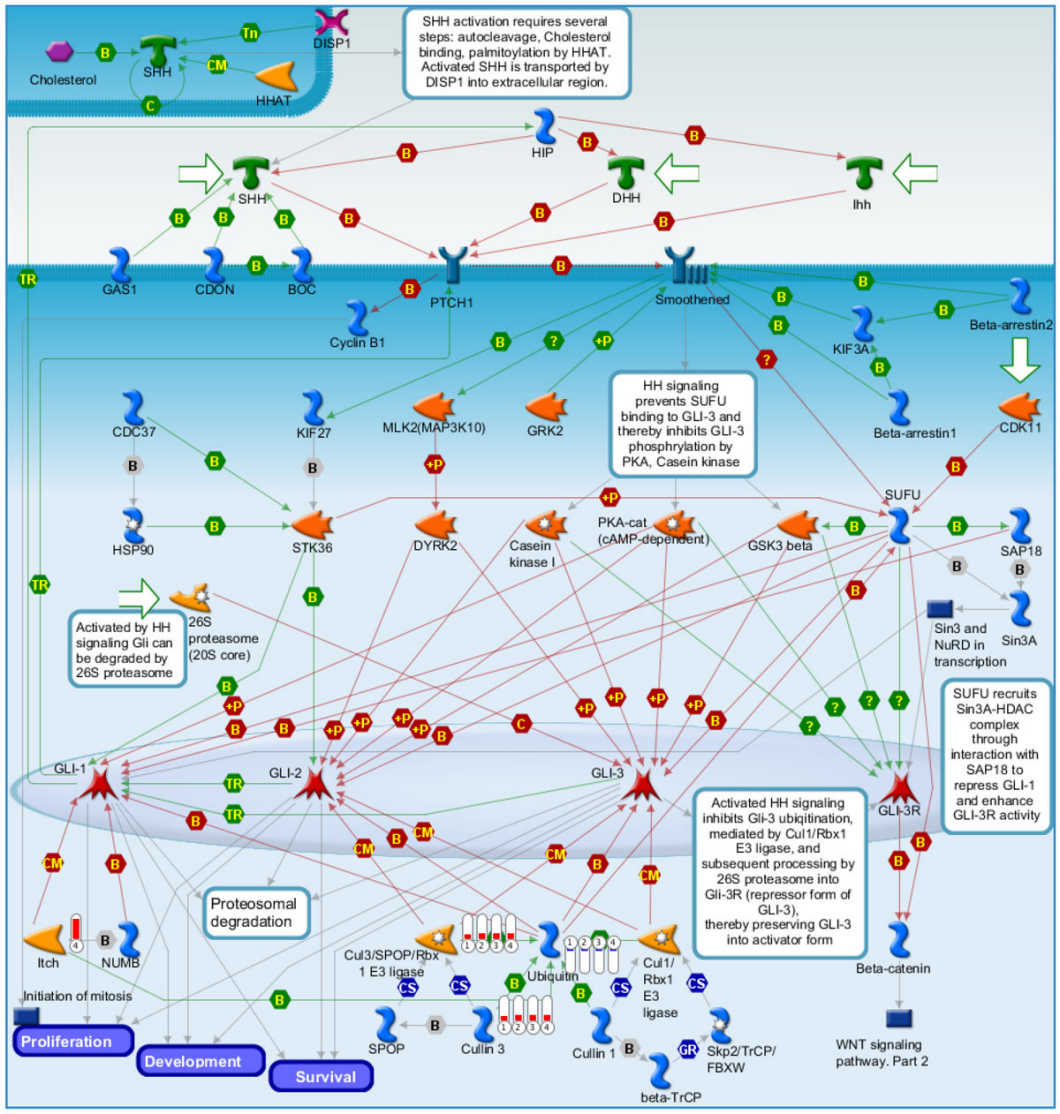
**Development Hedgehog Signaling which is the top scored pathway map in MetaCore enrichment analysis results**. The family of protein called Hedgehog controls and patterns various aspects of the vertebrate body plan such as survival, cell growth and etc. Ubiquitin was down-regulated while Cullin 3 and Cul3/SPOP/Rbx 1 E3 ligase complex was up-regulated in stoke samples at 3, 5, 24 hours and overall stroke samples as compared with control. ITCH was up-regulated in overall stroke samples. Figure 8-12: *Experimental data from all files is linked to and visualized on the maps as thermometer-like figures. Up-ward thermometers have red color and indicate up-regulated signals and down-ward (blue) ones indicate down-regulated expression levels of the genes. [The above paragraph is directly cited from the Metacore results.]

**Figure 9:**
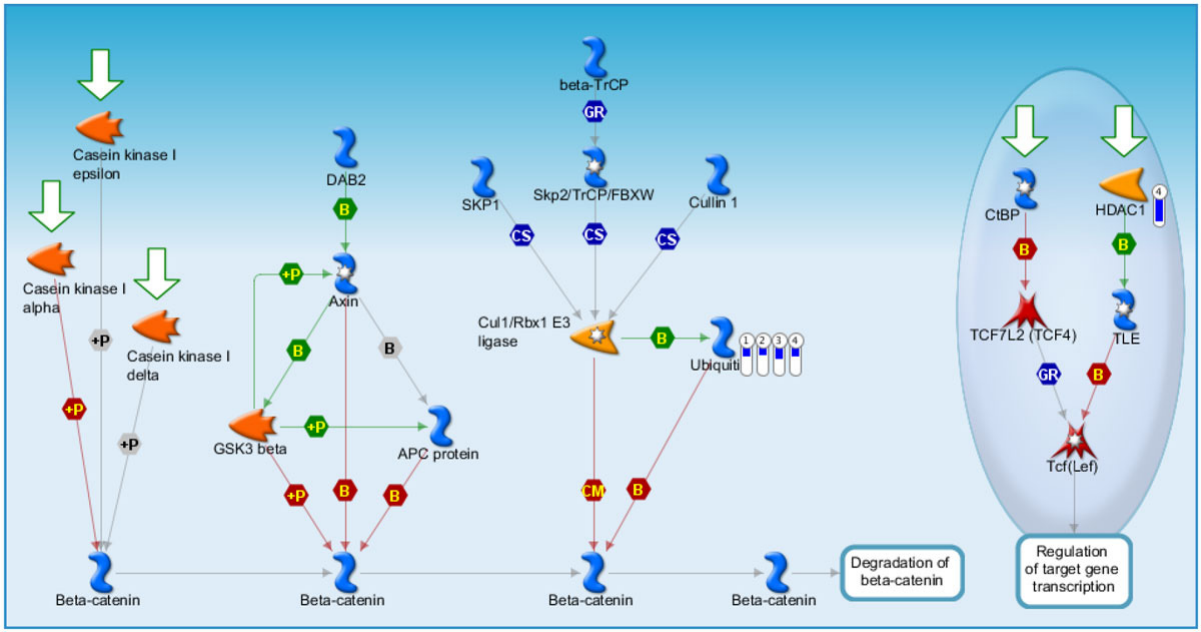
**Development WNT signaling pathway Part 1**. Degradation of beta catenin which is the second scored pathway map in MetaCore enrichment analysis results. Ubiquitin was down-regulated in stoke samples at 3, 5, 24 hours and overall stroke samples as compared with control. HDAC1 was down-regulated in overall stroke samples.

**Figure 10:**
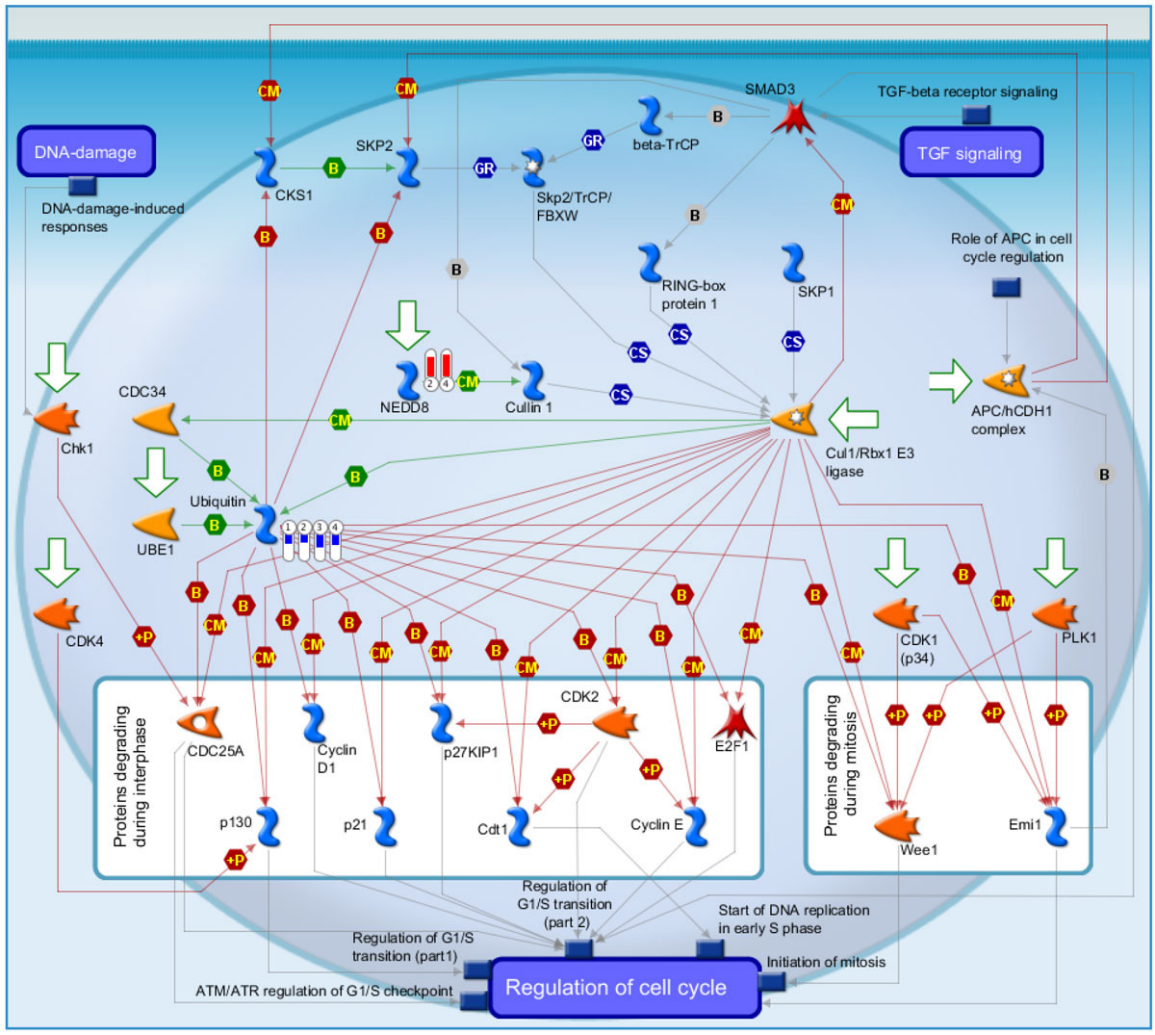
**Cell cycle Role of SCF complex in cell cycle regulation which is the third scored pathway map in MetaCore enrichment analysis results**. The Skp, Cullin, F-box containing complex (SCF complex) play critical roles in the ubiquitination of proteins involved in cell cycle regulation. Ubiquitin was down-regulated in stoke samples at 3, 5, 24 hours and overall stroke samples as compared with control. NEDD8 was up-regulated in stoke samples at 5 hours and overall stroke samples.

**Figure 11:**
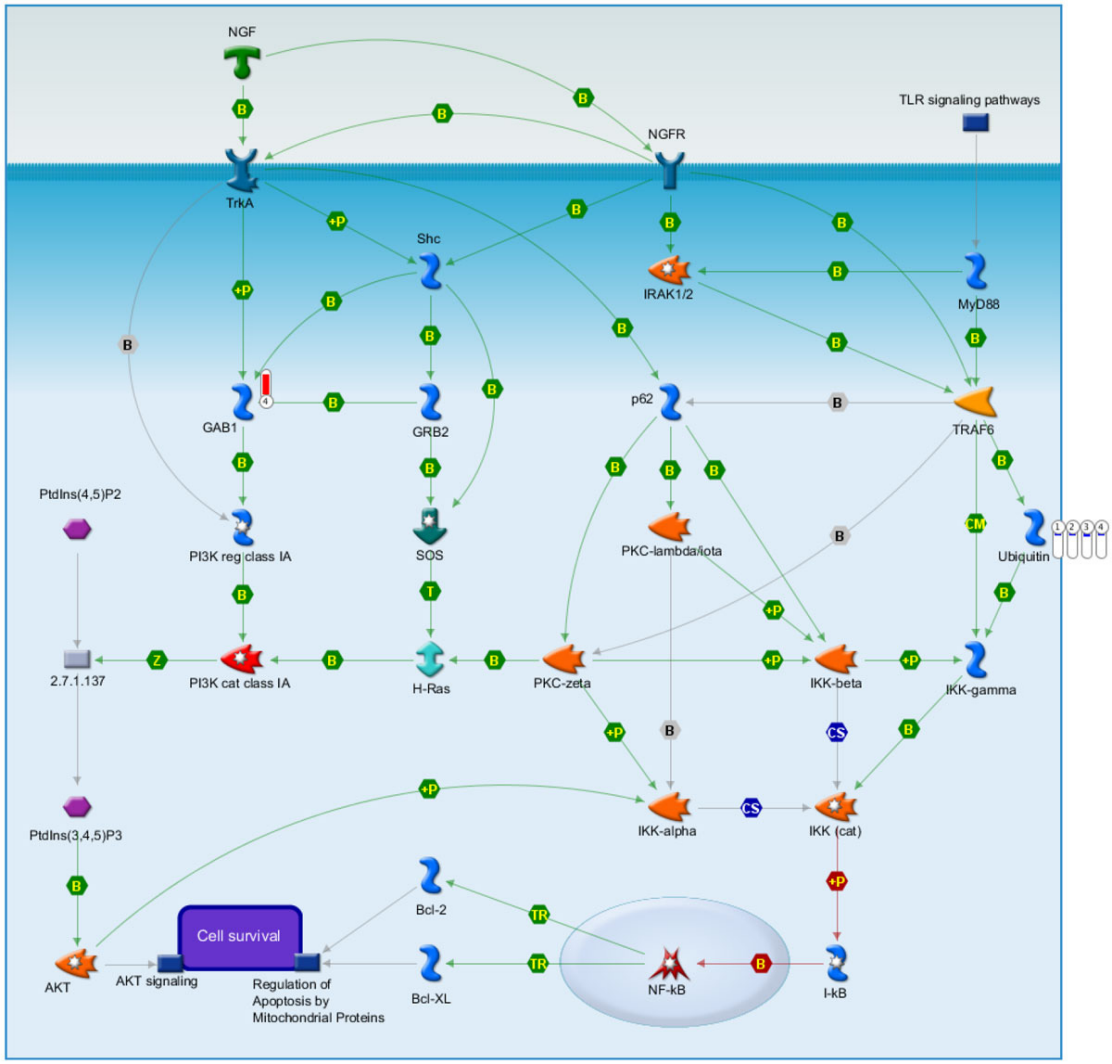
**Apoptosis and survival NGF activation of NF-kB which is the fourth scored pathway map in MetaCore enrichment analysis results**. Nerve growth factor (NGF) involved in neuron survival and differentiation, and the NF-kB signal generated by receptors of tyrosinekinase (TrkA) and the tumor necrosis factor receptor (NGFR) exerts neuroprotective effects. Ubiquitin was down-regulated in stoke samples at 3, 5, 24 hours and overall stroke samples as compared with control. GAB1 was up-regulated in overall stoke samples.

**Figure 12:**
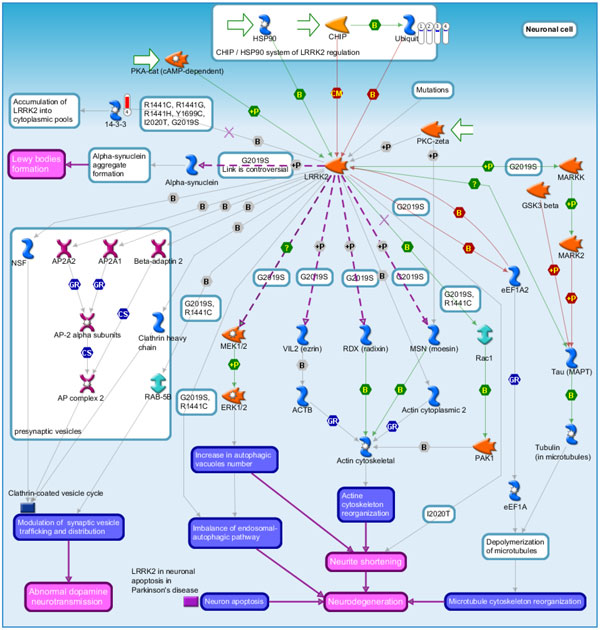
**LRRK2 in neurons in Parkinson's disease which is the fifth scored pathway map in MetaCore enrichment analysis results**. Mutation in LRRK2 (R1441C, R1441G, R1441H, Y1699C, I2020T and G2019S) are the most common genetic cause of Parkinson's disease, and LRRK2 stimulates various pathways leading to progression of Parkinson's disease. Ubiquitin was down-regulated in stoke samples at 3, 5, 24 hours and overall stroke samples as compared with control. LRRK2 was up-regulated in overall stroke samples.

**Figure 13:**
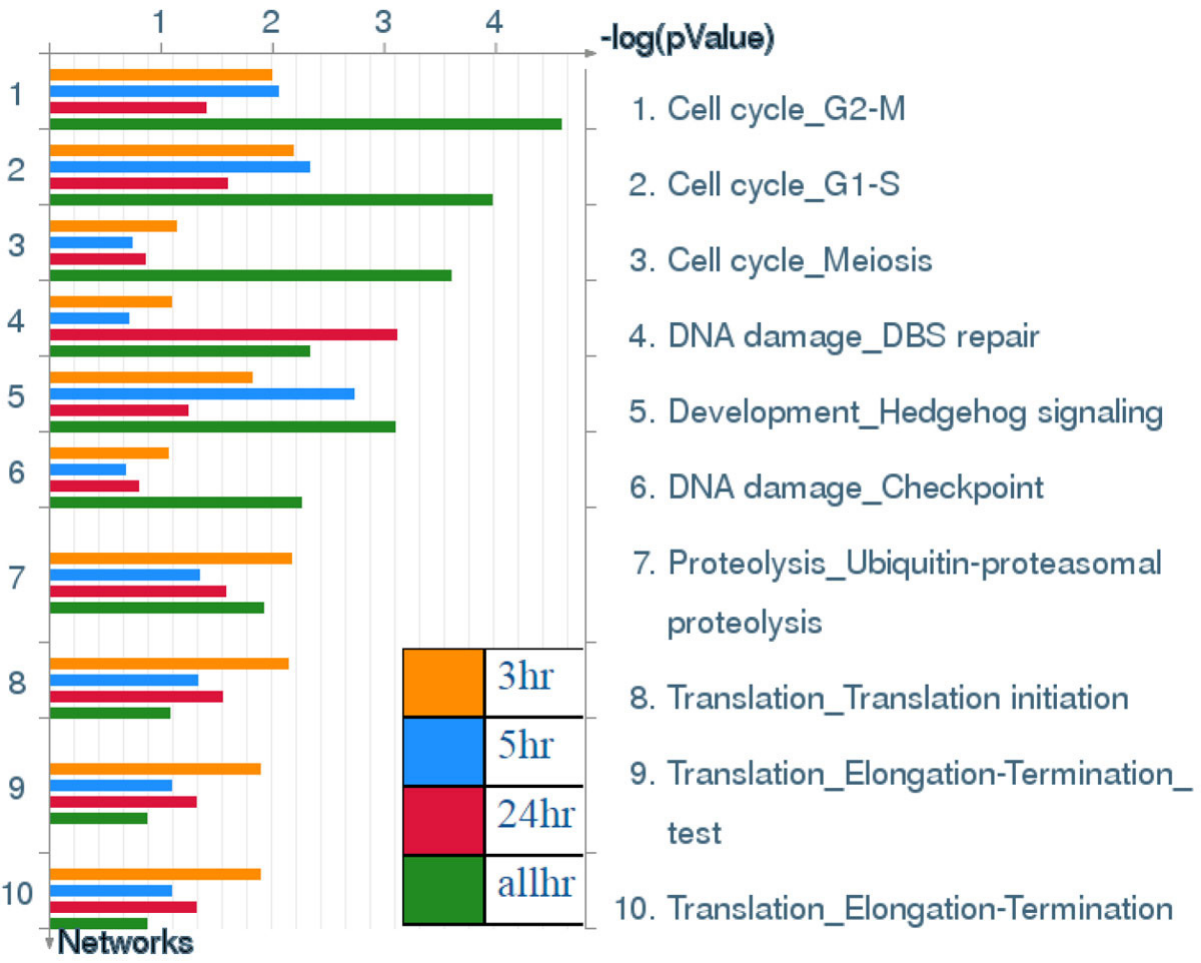
**Process Networks**. Sorting is done for the 'Statistically significant Networks'. The content of these cellular and molecular processes is defined and annotated by Thomson Reuters scientists. Each process represents a pre-set network of protein interactions characteristic for the process. [The above paragraph is directly cited from the Metacore results.]

**Figure 14:**
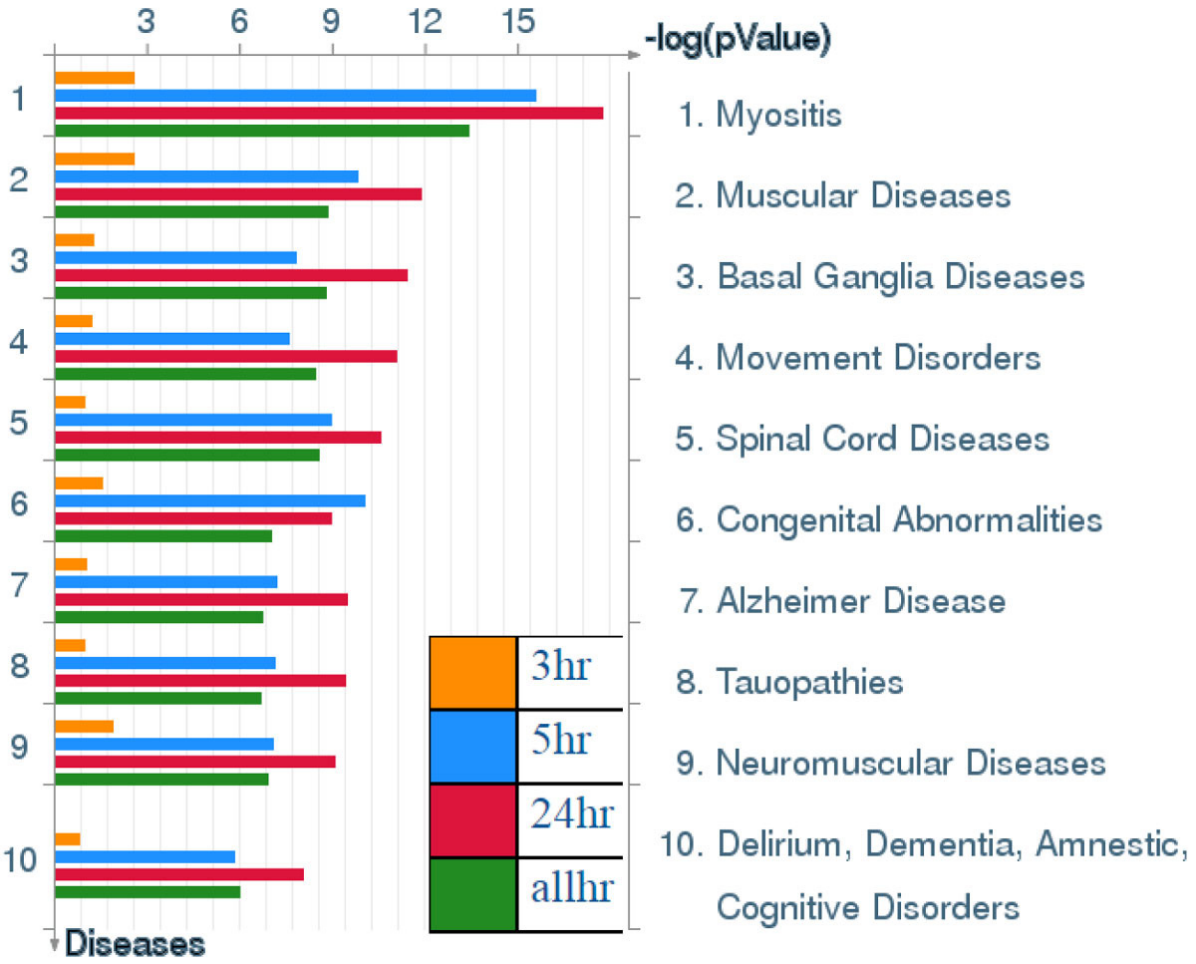
**Diseases (by Biomarkers)**. Sorting is done for the 'Statistically significant Diseases'. Disease folders are organized into a hierarchical tree. Gene content may very greatly between such complex diseases as cancers and some Mendelian diseases. Also, coverage of different diseases in literature is skewed. These two factors may affect p-value prioritization for diseases. [The above paragraph is directly cited from the Metacore results.]

IPA results (Figure 5) (Figure 6 shows the detailed legend of IPA in Figure 3, 4, 5) show that there were 6 modules of ML_Cell-Cycle-Brain, ML_Cell-Death-Brain, Tx_Cardiac-Necrosis/Cell Death, Tx_Cardiac-Fibrosis, Tx_Cardiac-Hypertrophy, and ML_Cardiovascular-Disease related to our 4 significant proteins (Figure 2(C)). Another key protein, SIRT7, was found at this time point. We found that SIRT7 was related to 4 modules. Vakhrusheva et al. discussed how "SIRT7 increases stress resistance of cardiomyocytes and prevents apoptosis and inflammatory cardiomyopathy in mice." It is a member of the mammalian sirtuin family that consists of 7 genes, SIRT1~7. Its deficiency can cause the development of heart hypertrophy and inflammatory cardiomyopathy [30]. SIRT7 was discovered to be highly associated with ischemic stroke in our analytical results. Previous studies showed the roles of sirtuins in cell death. Increasing evidence has suggested that sirtuins play fundamental roles in a variety of biological processes, including cell death, inflammation, and energy metabolism. In addition, SIRT7 increases the stress resistance of cardiomyocytes and prevents apoptosis and inflammatory cardiomyopathy in mice. We list the disease functional analyses in Additional file 3. IPA results are shown in Table 7. NOA results are shown in Table 8. Results of Metacore are shown in Figure 7 to 14, for 3, 5, and 24 h and the total (the sum of all samples).

**Table 7:** Functional analyses of the network biomarker at 24 h post-stroke.

Name	*p *value	Ratio
**Top canonical pathways**		

Docosahexaenoic acid (DHA) signaling	7.74E-03	1/39
Neuroprotective role of THOP1 in Alzheimer's disease	7.94E-03	1/40
Amyloid processing	1.01E-02	1/51
GABA receptor signaling	1.33E-02	1/67
Renal cell carcinoma signaling	1.41E-02	1/71

**Top disease and bio functions**		

Cancer	1.99E-04 ~ 5.96E-03	
Cardiovascular disease	1.99E-04 ~ 8.53E-03	
Connective tissue disorders	1.99E-04 ~ 5.97E-04	
Developmental disorder	1.99E-04 ~ 8.53E-03	
Hematological disease	1.99E-04 ~ 1.19E-03	

**Physiological system development and function**		

Behavior	1.99E-04 ~ 7.54E-03	
Cardiovascular system development and function	1.99E-04 ~ 6.55E-03	
Connective tissue development and function	1.99E-04 ~ 7.94E-03	
Embryonic development	1.99E-04 ~ 5.96E-03	
Hematological system development and function	1.99E-04 ~ 8.93E-03	

**Table 8:** The pathway analysis and gene set enrichment analysis of 4 proteins at 24 h post-stroke on (1) biological processes, (2) cellular components and (3) molecular functions by NOA.

GO: term	*p *value	Corrected *p *value	R	T	G	O	Term name
**(1) Biological Processes**							

GO:0042787	0.0033	0.0825	6357	1	21	1	protein ubiquitination involved in ubiquitin-dependent protein catabolic process
GO:0016567	0.0135	0.3382	6357	1	86	1	protein ubiquitination
GO:0032446	0.0171	0.4286	6357	1	109	1	protein modification by small protein conjugation
GO:0070647	0.0215	0.5387	6357	1	137	1	protein modification by small protein conjugation or removal
GO:0019941	0.0272	0.6803	6357	1	173	1	modification-dependent protein catabolic process
GO:0006511	0.0272	0.6803	6357	1	173	1	ubiquitin-dependent protein catabolic process
GO:0051603	0.0289	0.7236	6357	1	184	1	proteolysis involved in cellular protein catabolic process
GO:0043632	0.03	0.7511	6357	1	191	1	modification-dependent macromolecule catabolic process
GO:0006508	0.047	1	6357	1	299	1	proteolysis

**(2) Cellular Components**							

GO:0031463	7.8E-4	0.0110	6357	1	5	1	Cul3-RING ubiquitin ligase complex
GO:0031461	0.0042	0.0594	6357	1	27	1	cullin-RING ubiquitin ligase complex
GO:0000151	0.0099	0.1387	6357	1	63	1	ubiquitin ligase complex

**(3) Molecular Functions**							

GO:0031625	0.0020	0.0224	6357	1	13	1	ubiquitin protein ligase binding
GO:0019899	0.0056	0.0622	6357	1	36	1	enzyme binding
GO:0004842	0.0133	0.1470	6357	1	85	1	ubiquitin-protein ligase activity
GO:0019787	0.0143	0.1574	6357	1	91	1	small conjugating protein ligase activity
GO:0016881	0.0157	0.1730	6357	1	100	1	acid-amino acid ligase activity
GO:0016879	0.0212	0.2336	6357	1	135	1	ligase activity, forming carbon-nitrogen bonds
GO:0016874	0.0309	0.3408	6357	1	197	1	ligase activity

R: number of genes in the reference set. T: number of genes in the test set. G: number of genes annotated by a given term in the reference set. O: number of genes annotated by a given term in the test set.

### Network biomarkers and the evolution of network biomarkers of stroke etiology and repair processes

Our stroke PPI model was constructed from differential expressions of stroke and normal microarray data and data mining of PPI information from the BioGRID database. So, the 3 SPPINs and NPPIN were the results of our systems biology model using the original microarray data and PPI databases. There are 3 key factors which affected the final results.

**(i) The effect of different microarray data: **We know that microarray data have the drawback of being irreproducible. That means even in the same case, microarray data might not produce the same results as previous ones. Also, for the same diseases, patients of different ethnicities, different ages, or different genders will produce different microarray data. This is the first factor that affected the final results.

**(ii) The effect of different original PPI databases**: We know that PPI databases, such as BioGRID and MIPS, are constructed from putative information and then validated by wet-lab experiments. Due to advances in many high-throughput experimental skills, the original PPI databases have evolved with time. Newly updated original PPI databases were the second factor that affected the final results.

**(iii) The effect of the systems biology model: **Our mathematical model combined with many biological databases to be a novel one that we have successfully applied it on various cancer researches [9,10]. We used AIC and Student's *t*-test methods to construct the DPPIN of SPPIN and NPPIN, and get the SRV for three time points post stroke. The significance and the novelty of our model please refer to our previous work [9]. Although we described the novelty of our systems biology method, we have validated our results through a literature survey in the research. In the future, our results should be validated by other researchers' wet-lab experiments, and we will repeatedly modify our mathematical model. This is the third key factor that affected the results. Although not directly, it also had an influence on the protein interaction networks.

We also know that bio-systems evolve with time. It is obvious that different-stage patients have very different symptoms; these are key features for us to classify stroke stages. Since patients of different stages have greatly different symptoms, there is no doubt that the microarray data of these stage patients will be quite different. As described above, protein expressions from microarray data are one of the key factors of our systems biology model used to produce the final SPPINs and NPPIN. And the SPPINs and NPPIN yielded the final network biomarkers from our systems biology method. So, the most important thing for the evolution of network biomarkers is the evolution of microarray data at different stroke stages, which is inherent in the exhibition of stroke-related genes due to DNA mutations in the stroke process. The main purpose of this research was to discuss the network evolutionary process of stroke at 3 time points, and we hope it can provide clues for therapy and medical recovery processes. We found that CUL3 appeared at all 3 time points, and may be a target we should pay more attention to. At the second time point of 5 h, we found that the APP and caspase both played significant roles. At the last time point of 24 h, we found another important one, SIRT7. A lot of research has discussed these key proteins (Table 2).

Results in Figure 13 show that stroke-associated biomarker genes among different time points were significantly involved in cell cycle processing, including G_2_-M, G_1_-S and meiosis. Both in vitro and in vivo evidences for involvement of cell cycle elements in stroke was reported in a previous study [31]. The activity level of key regulators of the cell cycle are downregulated in differentiated neurons, and there is increasing evidence that activation of cell cycle machinery leads to death of neurons following stroke insults [32,33]. Our finding also shows the involvement of multiple cell cycle-regulatory signals in ischemic injury, and this may contribute to our current understanding of the etiology of stroke [34].

### Comparison with our previous results of traumatic brain injury in *Danio rerio*

We compared the results with our previous study, "On the Crucial Cerebellar Wound Healing-Related Pathways and Their Cross-Talks after Traumatic Brain Injury in *Danio rerio *[35]". We found that there were no intersections between these 2 results. To discuss core and specific network biomarkers of cardiac and brain injury between humans and other species is important work, and we will extend this work in the future. It is difficult to obtain datasets for stroke patients. The original reason we wanted to compare the results with traumatic brain injury in D. rerio was to determine if any intersection existed between these 2 results. Then maybe it would be possible to use D. rerio as a model organism to model human stroke. However, we found nothing at this stage, and we will try to develop other methods to model human stroke.

### Summary of results and discussion

Due to the help of high-throughput data and the power of our systems biology model, we determined total different network structures and biomarkers at 3 significant time points. Besides the original results of our model of SRV and network structure, we offer an abundant pathway analysis by various powerful commercial software and free web-servers. The entire work should be very valuable for experts (doctors and researchers) in developing novel strategies of recovery, therapy and prevention for stroke patients. Take for example, if you are only interested on SRVs, you can refer to Table 2 to choose the top SRV for drug targets. If you want to separate the PPIN by multiple drug targets, you can refer to Figure 1 to focus on elements of the network and select some of them to be drug targets. If you want to break down the network by destroying the regulatory relationship, you can refer to Figure 3 to 5, the IPA results, to choose some regulatory elements for your drug targets. If you want to break down the network by the complex modules given by Metacore, you can refer to Figure 7 to 14. You can use your medical knowledge combined with the complex modules to develop novel strategies. Additionally, the diseases and functional annotation given by IPA was shown in Additional file 4. And we also extended our research to examine relationships between significant genes determined by our models and many other diseases. This can give clues for new clinical application of old drug.

## Conclusions

Stroke is a complex disease, and its complex cellular behaviors differ from those of cancers. We found a lot of research work that focused on cancer systems biology, and not as much work on stroke systems biology. Our systems biology method applied to cancers helped us successfully identify network biomarkers. This is our first attempt to apply a similar framework of systematic theory to the stroke process. We focused on a systematic analysis of 3 key post-stroke time points, and our findings showed that stroke-associated biomarker genes among different time points were significantly involved in cell cycle processing, including G_2_-M, G_1_-S and meiosis, which contributes to our current understanding of the etiology of strokes. We identified a significant PPIN and the corresponding network biomarkers for 3 time points. We hope this work helps scientists reveal more hidden cellular mechanisms of stroke etiology and recovery processes. In future work, we will try to integrate more data samples and more critical time points of data, and design new methods of model organisms to unearth more deeply the mechanisms and processes.

## Competing interests

The authors declare that they have no competing interests.

## Authors' contributions

BSC and THC directed the research project. THC accumulated and organized the source data. YHW and CCW performed the experiments. YHW, CCW, and THC drafted different sections of the manuscript. HYL and HYW contributed viewpoints of clinical doctors to this research. BRJ helped to revise the manuscript. All of the authors approved publication of the manuscript.

## Supplementary Material

Additional file 1 The detailed description of Materials and Methods (*.pdf). Click here for file

Additional file 2 Parameter identification of the regression model in equation (1) by the maximum-likelihood method (*.pdf). Click here for file

Additional file 3 Determination of significant protein associations by the Akaike information criterion and Student's *t*-test (*.pdf). Click here for file

Additional file 4 **The diseases and functional annotation from IPA**. (*.zip). Click here for file

## Declarations

Publication costs for this article were funded by the Ministry of Science and Technology through grant nos. MOST-103-2745-E-007-001-ASP, MOST-103-2221-E-038 -013-MY2 and MOST 104-2218-E-007-021.

This article has been published as part of *BMC Systems Biology *Volume 9 Supplement 6, 2015: Joint 26th Genome Informatics Workshop and 14th International Conference on Bioinformatics: Systems biology. The full contents of the supplement are available online at http://www.biomedcentral.com/bmcsystbiol/supplements/9/S6.
