# Supplementary material for: Identification of network-based biomarkers of cardioembolic stroke using a systems biology approach with time series data
Source: BMC Syst Biol. 2015 Dec 9;9(Suppl 6):S4. doi: 10.1186/1752-0509-9-S6-S4 (PMC4674888; doi:10.1186/1752-0509-9-S6-S4)
Supplement: Additional file 1 — The detailed description of Materials and Methods (*.pdf). [file 1752-0509-9-S6-S4-S1.pdf]

Additional file 1: The detailed description of Materials and Methods

We have successfully used this similar theoretical framework to identify network biomarkers for various cancers. We put the full detailed description of the framework h ere.

A. Overview of the construction process of stroke network marker

We successfully used our methods to find the core and specific network markers of 4 different cancers and the evolution of network markers from the early to late stages of bladder cancer [9, 10]. A similar theoretical framework was employed in this study to find the evolution of network biomarkers of stroke at 3 time points which represent 3 important stages after a stroke has occurred. The theoretical systematic method in this paper was developed from a previous study. Figure 1 shows the flowchart to identify network biomarkers of stroke at 3 time points. We combined 2 kinds of data sources: (1) microarray data of stroke and normal samples from the Gene Expression Omnibus (GEO) database where stroke samples are divided into 3 groups according to the time points of 3, 5, and 24 h post-stroke; and (2) a PPI database, which was required to construct 3 candidate PPINs of stroke. Microarray data were used for PPI pool selection, and then the selected PPIs and expression profiles of differentially expressed genes were used to construct a PPIN. Through regression modeling and a maximum-likelihood parameter estimation method, 3 stroke PPINs (SPPINs) and a normal PPIN (NPPIN) were then obtained. We also created differential PPINs (DPPINs) by comparing the constructed SPPINs with the NPPIN. In the DPPINs, a set of significant proteins for stroke were obtained as network markers at each time point (stage) based on the stroke relevance value (SRV) of each protein in equation (8) and a statistical assessment described in subsection D of the “Materials and methods” section.

B. Data selection and pre-processing

The stroke microarray dataset was obtained from the NCBI GEO [11]. In this study, we chose GSE58294 [12] and its corresponding platform, GPL570, as our research object (Table 1). It contains gene expression data following a cardioembolic stroke. We only used data derived from non-processed primary biopsies to avoid discrepancies in gene expressions that are intrinsic to cell culture and fixation. Therefore, the dataset contained 3 time points of stroke patients and 23 control samples from non-disease subjects. We built SPPINs for 3, 5, and 24 h post-stroke in this study and the NPPIN. The dataset contained 23 samples for each stage. Prior to further analysis, the gene expression value, h_{ij}, of gene i in the jth sample was normalized to z-transformed scores, g_{ij}, and then the resultant normalized expression value had a mean μ_i=0 and standard deviation σ_i=1 over sample j [13, 14].

PPI data for *Homo sapiens* were extracted from the Biological General Repository for Interaction Database (BioGRID). The BioGRID is an open-access archive of genetic and protein interactions that are curated from the primary biomedical literature of all major model organisms [15]. BioGRID was mined for candidate stroke PPINs which were pruned to delete false-positive PPIs using their corresponding microarray data. These PPINs of 3, 5, and 24 h post-stroke and normal stages were then compared to obtain network markers.

C. Selection of a protein pool and identification of PPINs for normal and stroke stages

To integrate gene expressions with PPI data to construct the corresponding SPPINs and NPPIN, we set up a protein pool containing differentially expressed proteins. Gene expression values were reasonably assumed to correlate with protein expression levels. We used a one-way analysis of variance (ANOVA) to analyze the

expression of each protein and select for proteins with significant differential expression levels. This method allowed determination of significant differences between the stroke and normal datasets. The null hypothesis (Ho) was based on the assumption that mean protein expression levels of stroke and normal sets were the same. The Bonferroni adjustment [16], a type of correction for multiple testing, was used to detect proteins with a discrepancy. Proteins with a p value of <0.01 were included in the protein pool, while proteins in the protein pool with no PPI information were eliminated. In addition, proteins that were not already in the protein pool were included if their PPI information indicated that they had a close relationship with a protein already in the pool. As a result, the protein pool contained proteins that had significant differences in expression levels and proteins that had close relationships with the aforementioned proteins.

On the strength of the significant protein pool and PPI information, candidate PPINs for 3, 5, and 24 h post-stroke and normal stages were constructed by linking proteins that interacted with each other. In other words, proteins that had PPI information through the pool were linked together, resulting in candidate PPINs.

As the candidate PPIN included all possible PPIs under various environments and experimental conditions, the candidate PPIN needed to be further confirmed by microarray data to identify appropriate PPIs according to the biological processes that are relevant to stroke. To remove false-positive PPIs from each candidate PPIN for different biological conditions, we used both a PPI model identification scheme and a model order detection method to prune each candidate PPIN using corresponding microarray data to approach the actual PPIN of stroke. Here, the PPI of target protein i in the candidate PPIN can be depicted by the following protein association model:

$$x_i(n) = \sum_{j=1}^{M_i} \alpha_{ij} x_j(n) + \omega_i(n) \quad (1)$$

where $x_i[n]$ is the expression levels of target protein i for sample n; $x_j[n]$ is the expression level of the j-th protein interacting with target protein i for sample n; α_{ij} is the association interaction ability between target protein i and its j-th interactive protein; M_i is the number of proteins interacting with target protein i; and $\omega_i[n]$ is stochastic noise due to other factors or model uncertainty. The biological meaning of equation (1) is that expression levels of target protein i are associated with expression levels of proteins that interact with it. Consequently, a protein association (interaction) model for each protein in the protein pool can be built using equation (1).

After constructing equation (1) for the PPI model of each protein in the candidate PPIN, we used the maximum-likelihood (ML) estimation method [17] to identify associated parameters in (1) by microarray data as follows (see Additional file S.1):

$$x_i(n) = \sum_{j=1}^{M_i} \hat{\alpha}_{ij} x_j(n) \quad (2)$$

where $\hat{\alpha}_{ij}$ was identified using microarray data in accordance with the ML estimation method.

Once association parameters for all proteins in the candidate PPIN were identified for each protein, significant protein associations were determined using the model order detection method based on the estimated association abilities, i.e., detecting the interaction number, M_i, in equation (2). The Akaike information criterion (AIC) [17] and a Student's t-test [18] were used for both model order selection and significance determination of protein associations in $\hat{\alpha}_{ij}$ (see Additional file S.2).

D. Determination of significant proteins and their network structures at 3, 5, and 24 h post-stroke and normal cells

After the interaction number, M_i', was determined using the AIC order detection and Student's *t*-test, spurious false-positive PPIs, $\hat{\alpha}_{ij}$, in equation (2) were pruned away, and only significant PPIs that remained were refined as follows:

$$x_i(n) = \sum_{j=1}^{M_i'} \hat{\alpha}_{ij} x_j(n),\ i = 1,2......M \tag{3}$$

where $M_i' \leq M_i$ is the number of significant PPIs in the PPIN, with target protein *i*. In other words, a number of $M_i' \leq M_i$ (or false positives) were pruned from the PPIs of target protein *i*. Protein by protein (i.e., $i = 1,2,...,M$ for all proteins in equation (3)) pruning resulted in the following refined PPIN:

$$X(n) = AX(n) + w(n) \tag{4}$$

where

$$X(n) = \begin{bmatrix} x_1(n) \\ x_2(n) \\ \vdots \\ x_M(n) \end{bmatrix},\quad A = \begin{bmatrix} \hat{\alpha}_{11} & \cdots & \hat{\alpha}_{1M} \\ \vdots & \ddots & \vdots \\ \hat{\alpha}_{M1} & \cdots & \hat{\alpha}_{MM} \end{bmatrix}, \text{ and } \quad w(n) = \begin{bmatrix} w_1'(n) \\ w_2'(n) \\ \vdots \\ w_M'(n) \end{bmatrix}.$$

The interaction matrix *A* denotes PPIs in their refined PPIN. If there was no PPI between proteins *i* and *j* or it was pruned away by AIC order detection due to insignificance in the refined PPIN then $\hat{\alpha}_{ij}=0$. In general, $\hat{\alpha}_{ij}=\hat{\alpha}_{ji}$, but if that was not the case, the larger one was chosen as $\hat{\alpha}_{ij}=\hat{\alpha}_{ji}$ to avoid a situation where $\hat{\alpha}_{ij} \neq \hat{\alpha}_{ji}$. The above PPIN construction method was employed to construct refined PPINs for 3, 5, and 24 h post-stroke and normal cells. The interaction matrix *A* of refined PPINs in equation (4) for 3, 5, and 24 h post-stroke and normal cells was constructed, respectively, as follows:

$$A_S^k = \begin{bmatrix} \hat{\alpha}_{11,S}^k & \cdots & \hat{\alpha}_{1M,S}^k \\ \vdots & \ddots & \vdots \\ \hat{\alpha}_{M1,S}^k & \cdots & \hat{\alpha}_{MM,S}^k \end{bmatrix}, \text{ and } A_N = \begin{bmatrix} \hat{\alpha}_{11,N} & \cdots & \hat{\alpha}_{1M,N} \\ \vdots & \ddots & \vdots \\ \hat{\alpha}_{M1,N} & \cdots & \hat{\alpha}_{MM,N} \end{bmatrix} \quad (5)$$

where k = 3, 5, and 24 h post-stroke; A_S^k and A_N are interaction matrices of the refined PPINs of 3, 5, and 24 h post-stroke, respectively; and M is the number of proteins in the refined PPIN. Therefore, the protein association model for SPPINs and the NPPIN for 3, 5, and 24 h post-stroke and normal cells can be represented by the following equations according to equations (4) and (5):

$$\begin{aligned} x_S^k(n) &= A_S^k x_S(n) \\ x_N(n) &= A_N x_N(n) \end{aligned} \quad (6)$$

where k = 3, 5, and 24 h post-stroke and $x_S^k(n) = \begin{bmatrix} x_{1S}^k & x_{2S}^k & \cdots & x_{MS}^k \end{bmatrix}^T$ and $x_N(n)$=[x_{1N} x_{2N} ··· x_{MN}]^T are vectors of expression levels. The difference matrix $A_S^k - A_N$ of the DPPIN between SPPINs and NPPIN is defined as follows:

$$D^k = \begin{bmatrix} d_{11}^k & \cdots & d_{1M}^k \\ \vdots & \ddots & \vdots \\ d_{M1}^k & \cdots & d_{MM}^k \end{bmatrix} = \begin{bmatrix} \hat{\alpha}_{11,S}^k - \hat{\alpha}_{11,N} & \cdots & \hat{\alpha}_{1M,S}^k - \hat{\alpha}_{1M,N} \\ \vdots & \ddots & \vdots \\ \hat{\alpha}_{M1,S}^k - \hat{\alpha}_{M1,N} & \cdots & \hat{\alpha}_{MM,S}^k - \hat{\alpha}_{MM,N} \end{bmatrix}; \quad (7)$$

where k = 3, 5, and 24 h post-stroke; d_{ij}^k is the protein association ability difference between SPPINs and NPPIN at k = 3, 5, and 24 h post-stroke and normal cells; and matrix D^k is the difference in network structures between SPPINs and the NPPIN for k = 3, 5, and 24 h post-stroke and normal cells. In order to investigate stroke-related factors from the difference matrix, D^k, between SPPINs and the NPPIN at 3, 5, and 24 h post-stroke and normal cells in equation (7), a score, which we named the SRV, is presented to quantify the correlation of each protein in D^k with the significance of stroke as follows [13]:

$$SRV^k = \begin{bmatrix} SRV_1^k \\ \vdots \\ SRV_i^k \\ \vdots \\ SRV_M^k \end{bmatrix} \quad (8)$$

where $SRV_i^k = \sum_{j=1}^{M} \left| d_{ij}^k \right|$, and $k = 3$, 5, and 24 h post-stroke. The SRV_i^k in equation (8) quantifies the differential extent of protein associations of the i-th protein (the absolute sum of the i-th row of D^k in equation (7)) and the SRV_i^k can differentiate the SPPIN from the NPPIN in the k-th stage of a stroke. In other words, the SRV_i^k in equation (8) represents the network structural difference of the i-th protein between the stroke and normal networks in the k-th stage of stroke.

In order to investigate what proteins are more likely involved in the k-th stage of a stroke, we needed to calculate the corresponding empirical p value to determine the statistical significance of the SRV_i^k. To determine the observed p value of each SRV_i^k, we repeatedly permuted the network structure of the candidate PPINs at 3, 5, and 24 h post-stroke as a random network. Each protein in the random network at 3, 5, and 24 h post-stroke had its own SRV to generate a distribution of SRV_i^k for $k = 3$, 5, and 24 h post-stroke. Although there was a random disarrangement of the network structure, linkages of each protein were maintained. In other words, proteins with which a particular protein interacted were permuted without changing the total number of protein interactions. This procedure was repeated 10^5 times, and the corresponding p value was calculated as a fraction of the random network structure in which the SRV_i^k was at least as large as the SRV of the actual network structure.

According to the distributions of the SRV_i^k of random networks, the SRV_i^k in equation (8) with a p value of ≤0.01 was regarded as a significant *SRV*, and the corresponding protein was determined to be a significant protein at 3, 5, and 24 h post-stroke; a protein with a p value of >0.01 was removed from the list of significant proteins in stroke (in other words, if the p value of SRV_i^k was >0.01, then the i-th protein was removed from the SRV_i^k in equation (8) and the remainder of the SRV_i^k with p values of *SRVs* of <0.01 were considered significant proteins at 3, 5, and 24 h post-stroke).

Based on the p value of the *SRVs* for all proteins (i=1, 2, ···, M) and the 3 stages of stroke (k = 3, 5, and 24 h post-stroke), we generated 2 lists of significant proteins for each of the 3 stages according to the *SRV* and the statistical assessment of each significant protein in SRV_i^k in equation (8). We also found 5, 9, and 4 significant proteins, respectively, as the specific network markers of 3, 5, and 24 h post-stroke. These proteins showed significant changes between SPPINs and the NPPIN in the stroke process according to the corresponding stroke stage, and we suspected that these changes might play important roles in the stroke process. These findings warrant further investigation.

E. Pathway analysis

More-valuable cellular information can be found in known pathways, which are useful for describing most "normal" biological phenomena. All of these known pathways are the result of repeated testing and verification, and the entire pathway network has definitions for most links. Therefore, proteins we identified to be significant in the above network markers were mapped onto known pathway networks (e.g., the Kyoto Encyclopedia of Genes and Genomes (KEGG) pathway) to

investigate significant pathways with the network markers and explore relationships between these pathways and stroke. This approach supports the view that systems biology can help identify significant network biomarkers in stroke and relate their cellular roles in stroke etiology and repair processes.

Together with comprehensive pathway databases such as the KEGG, we used a series of bioinformatics pathway analytical tools to identify biologically relevant pathway networks. The KEGG includes manually curated biological pathways that cover 3 main categories: systems information (e.g., human diseases and drugs), genomics information (e.g., gene catalogs and sequence similarities), and chemical information (e.g., metabolites and biochemical reactions). At present, the KEGG contains 134,511 distinct pathways generated from 391 original reference pathways [19]. Therefore, to investigate the pathways involved in stroke etiology and repair processes, the DAVID bioinformatics database [20, 21], which generates automatic outputs of the results from a KEGG pathway analysis [22], was used for the pathway analysis of significant proteins identified in network markers to determine their cellular roles in stroke.

To complete our research results, we used the well-known commercial software, Ingenuity® Pathway Analysis (IPA), to do multiple functional and pathway analyses. IPA® is from QIAGEN (Redwood City, CA, www.qiagen.com/ingenuity). We then used free network ontology analysis (NOA) software to do the pathway analysis and gene set enrichment analysis (GSEA) on biological processes, cellular components, and molecular functions [23]. The NOA first defines link ontology that assigns functions to interactions based on the known annotations of joint genes via optimizing 2 novel indexes 'Coverage' and 'Diversity'. Then, the NOA generates 2 alternative reference sets to statistically rank the enriched functional terms for a given biological network. Wang *et al.* compared NOA with traditional enrichment analysis methods in

several biological networks, and found that: (i) the NOA can capture changes in functions not only in dynamic transcription regulatory networks but also in rewiring protein interaction networks while the traditional methods cannot and (ii) the NOA can find more relevant and specific functions than traditional methods in different types of static networks. The above description of NOA is directly cited from their paper [24].
