# Supplementary material for: Identification of network-based biomarkers of cardioembolic stroke using a systems biology approach with time series data
Source: BMC Syst Biol. 2015 Dec 9;9(Suppl 6):S4. doi: 10.1186/1752-0509-9-S6-S4 (PMC4674888; doi:10.1186/1752-0509-9-S6-S4)
Supplement: Additional file 2 — Parameter identification of the regression model in equation (1) by the maximum-likelihood method (*.pdf). [file 1752-0509-9-S6-S4-S2.pdf]

Additional file 2

Parameter Identification of Regression Model in Equation (1) by Maximum Likelihood Method

Equation (1) can be written as the following requiring form

$$x_i[n] = \left[x_1[n] \ \cdots \ x_{Mi}[n]\right] \begin{bmatrix} \alpha_{i1} \\ \vdots \\ \alpha_{iM_i} \end{bmatrix} + \omega_i[n] = \phi_i[n] \cdot \theta_i + \omega_i[n] \tag{S1}$$

where $\phi_i[n]$ denotes the regression vector which can be obtained from microarray data, θ_i is the parameter vector to be estimated. Suppose that there are *m* samples, then it is easy to acquired values of $\{x_i[n] \ \phi_i[n]\}$ for $n \in \{1, \ \ldots \ ,m\}$. In this case, equation (S1) for different samples can be represented as the following vector form.

$$\begin{bmatrix} x_i[1] \\ \vdots \\ x_i[m] \end{bmatrix} = \begin{bmatrix} \phi_i[1] \\ \vdots \\ \phi_i[m] \end{bmatrix} \cdot \theta_i + \begin{bmatrix} \omega_i[1] \\ \vdots \\ \omega_i[m] \end{bmatrix} \tag{S2}$$

where $\phi_i[m] = [x_i(m) \cdots x_{Mi}(n)], \theta_i = [\alpha_{i1} \cdots \alpha_{iM_i}]$

For simplicity, it can be represented as follows.

$$X_i = \Phi_i \cdot \theta_i + e_i \tag{S3}$$

where $e_i = [w_1(1) \cdots w_i(m)]^T$.

In equation (S3), the noise e_i for different samples was regarded as independent random variables of normal distribution with zero mean and unknown variance σ_i^2, i.e., $E\{e_i\} = 0$, and $\Sigma_i = E\left\{e_i e_i^T\right\} = \sigma_i^2 I$, where *I* is the identity matrix. The probability density function of e_i is given as follows.

$$p(e_i) = \frac{1}{((2\pi)^m \det \Sigma_i)^{1/2}} \exp(-\frac{1}{2} e_i^T \Sigma_i^{-1} e_i) \tag{S4}$$

From equation (S4), we can obtain the likelihood function

$$L(\theta_i, \sigma_i^2) = p(\theta_i, \sigma_i^2) = \frac{1}{(2\pi\sigma_i^2)^{m/2}} \exp(-\frac{(X_i - \Phi_i\theta_i)^T (X_i - \Phi_i\theta_i)}{2\sigma_i^2}) \tag{S5}$$

Maximum likelihood estimation method aims at finding θ_i and σ_i^2 to maximize the likelihood function in equation (S5). In order to simplify the computation, it is practical to take the logarithm of the likelihood function, which yields the following log-likelihood function:

$$\log L(\theta_i, \sigma_i^2) = -\frac{m}{2} \log(2\pi\sigma_i^2) - \frac{1}{2\sigma_i^2} \sum_{n=1}^{m} \left[y_i[n] - \phi_i[n] \cdot \theta_i \right]^2 \tag{S6}$$

where $x_i[n]$ and $\phi_i[n]$ are the *n*-th element of X_i and Φ_i in (S3), respectively.

By the maximum likelihood parameter estimation method, we expect the log-likelihood function to have the maximum at $\theta_i = \hat{\theta}_i$ and $\sigma_i^2 = \hat{\sigma}_i^2$. The necessary conditions for the maximum likelihood estimates $\hat{\theta}_i$ and $\hat{\sigma}_i^2$ must conform to the following two equations.

$$\begin{aligned} \frac{\partial \log L(\theta_i, \sigma_i^2)}{\partial \theta_i} &= 0 \\ \frac{\partial \log L(\theta_i, \sigma_i^2)}{\partial \sigma_i^2} &= 0 \end{aligned} \tag{S7}$$

The estimated parameters $\hat{\theta}_i$ and $\hat{\sigma}_i^2$ are shown below,

$$\hat{\theta}_i = (\Phi_i^T \Phi_i)^{-1} \Phi_i^T Y_i \tag{S8}$$

$$\hat{\sigma}_i^2 = \frac{1}{m} \sum_{n=1}^{m} \left[x_i[n] - \phi_i[n] \cdot \hat{\theta}_i \right]^2 = \frac{1}{m} (X_i - \Phi_i \cdot \hat{\theta}_i)^T (X_i - \Phi_i \cdot \hat{\theta}_i) \tag{S9}$$

where X_i and Φ_i can be obtained from the microarray in the rough PPIN. Since there are two data sets of microarray data, two association parameters for stroke and normal were separately identified.
